# Alternative translation and retrotranslocation of cytosolic C3 that detects cytoinvasive bacteria

**DOI:** 10.1007/s00018-022-04308-z

**Published:** 2022-05-11

**Authors:** Mariann Kremlitzka, Lucie Colineau, Alicja A. Nowacka, Frida C. Mohlin, Katarzyna Wozniak, Anna M. Blom, Ben C. King

**Affiliations:** 1 Division of Medical Protein Chemistry, Department of Translational Medicine, Lund University, Malmö, Sweden; 2 Department of Immunology, Eötvös Loránd University, Budapest, Hungary

**Keywords:** Complement factor C3, Cellular trafficking, Intracellular complement, Pathogen sensing

## Abstract

Complement C3 was originally regarded as a serum effector protein, although recent data has emerged suggesting that intracellular C3 can also regulate basic cellular processes. Despite the growing interest in intracellular C3 functions, the mechanism behind its generation has not been demonstrated. In this study we show that C3 can be expressed from an alternative translational start site, resulting in C3 lacking the signal peptide, which is therefore translated in the cytosol. In contrast to the secreted form, alternatively translated cytosolic C3 is not glycosylated, is present mainly in a reduced state, and is turned over by the ubiquitin–proteasome system. C3 can also be retrotranslocated from the endoplasmic reticulum into the cytosol, structurally resembling secreted C3. Finally, we demonstrate that intracellular cytosolic C3 can opsonize invasive *Staphylococcus aureus* within epithelial cell, slowing vacuolar escape as well as impacting bacterial survival on subsequent exposure to phagocytes. Our work therefore reveals the existence and origin of intracellular, cytosolic C3, and demonstrates functions for cytosolic C3 in intracellular detection of cytoinvasive pathogens.

**Supplementary Information:**

The online version contains supplementary material available at 10.1007/s00018-022-04308-z.

## Introduction

The complement system is classically viewed as a liver-derived serum effector cascade of innate immunity, with three activation pathways leading to the enzymatic cleavage of the central complement component, C3, into C3a and C3b. While C3a induces mobilization of immune cells and modulates inflammation, C3b is an opsonin and allows further propagation of the cascade in order to remove altered self and non-self structures [1]. Despite the well-recognized role of extracellular complement in immune defense, evidence is accumulating that many immune and non-immune cell types synthesize complement proteins [2] and these locally or even intracellularly acting complement components drive basic cellular processes, maintaining cellular and tissue homeostasis [3, 4]. These results have revolutionized the concept of complement [5] and initiated the era of intracellular complement.

Human C3 is encoded by a single gene, consisting of 41 exons [6]. C3 mRNA is translated by endoplasmic reticulum (ER)-associated ribosomes as pre-pro C3 containing a 22 amino acid signal peptide necessary for entry into the ER and the secretory pathway [7]. During ER entry, the signal sequence is removed, yielding pro-C3, a 180 kDa polypeptide, glycosylated on Asn63 and Asn917. In the Golgi network, C3 is further processed by an endoprotease of the furin family [8], generating mature C3, consisting of a 110 kDa α-chain and a 75 kDa β-chain [9], connected by a single disulphide bond. Secreted serum C3 is produced mainly in the liver [10], however C3 synthesis has also been reported in many other sites [11]. This local expression can be vital for the function of specific cell types. For example, the local expression and activation of complement proteins, resulting in auto- and paracrine stimulation via C3aR, has been shown to be important during the interaction of dendritic and T-cells [12, 13]. In addition to these canonical functions of locally expressed complement proteins, the recent concept of intracellular complement has also been introduced, with findings that intracellular activation of C3 can be important for cellular survival in T-cells [14] and lung epithelial cells [15]. Further work has shown that within the cell, C3 could regulate basic cellular processes such as autophagy [16–18], gene transcription [19] and metabolism [20]. The exact molecular mechanisms of these functions are still unclear, and the specific sub-cellular locations of the components involved still need to be elucidated, although C3 stores have been identified in lysosomes and late endosomes in T-cells [14] and lung epithelial cells [15] respectively. Where the local, i.e. cell-intrinsic source of C3 is known to be important for functions of intracellular C3, a question remains whether C3 is first secreted, and then re-internalized [21], or whether there is another or additional pathway whereby intracellular C3 is retained or diverted from the secretory pathway. In addition, interactions of C3 with partners found within the cytosol itself, the gel-like fluid filling the cell volume and in which organelles are suspended, have also been reported, including our findings that cell-intrinsic C3 regulates homeostatic autophagy via interaction with the cytosolic protein ATG16L1 [16]. How cell-intrinsic C3 could enter the cytosol to interact with these partners has remained unexplored until now.

In this study, we aimed to identify the mechanism for C3 entry into the cytosol, and to characterize intracellular C3 species. We show that beside the recently described internalization of extracellular C3 (endosomal C3) [19, 21], C3 is also present in the cytosol and can originate either from an alternative translational start site downstream of the signal peptide, or may be retrotranslocated from the ER. In addition, we demonstrate a functional relevance of cytosolic C3, by opsonization of cytoinvasive *Staphylococcus aureus* in cells that do not express canonical secretory C3. We describe a novel cytosolic C3 fragment that alters *S. aureus* phenotype during infection of lung epithelial cells and augments subsequent phagocytosis. The distinct cytosolic C3 forms may define separate functions and hence, our data may help to understand the difference between intracellular- and serum C3 deficiencies and might promote development of therapies to selectively inhibit the intracellular complosome in diseases in which the pathomechanism involves abnormal intracellular C3 function.

## Results

### Cytosolic C3 lacking a signal peptide is translated from non-canonical translation start sites

**Fig. 1:**
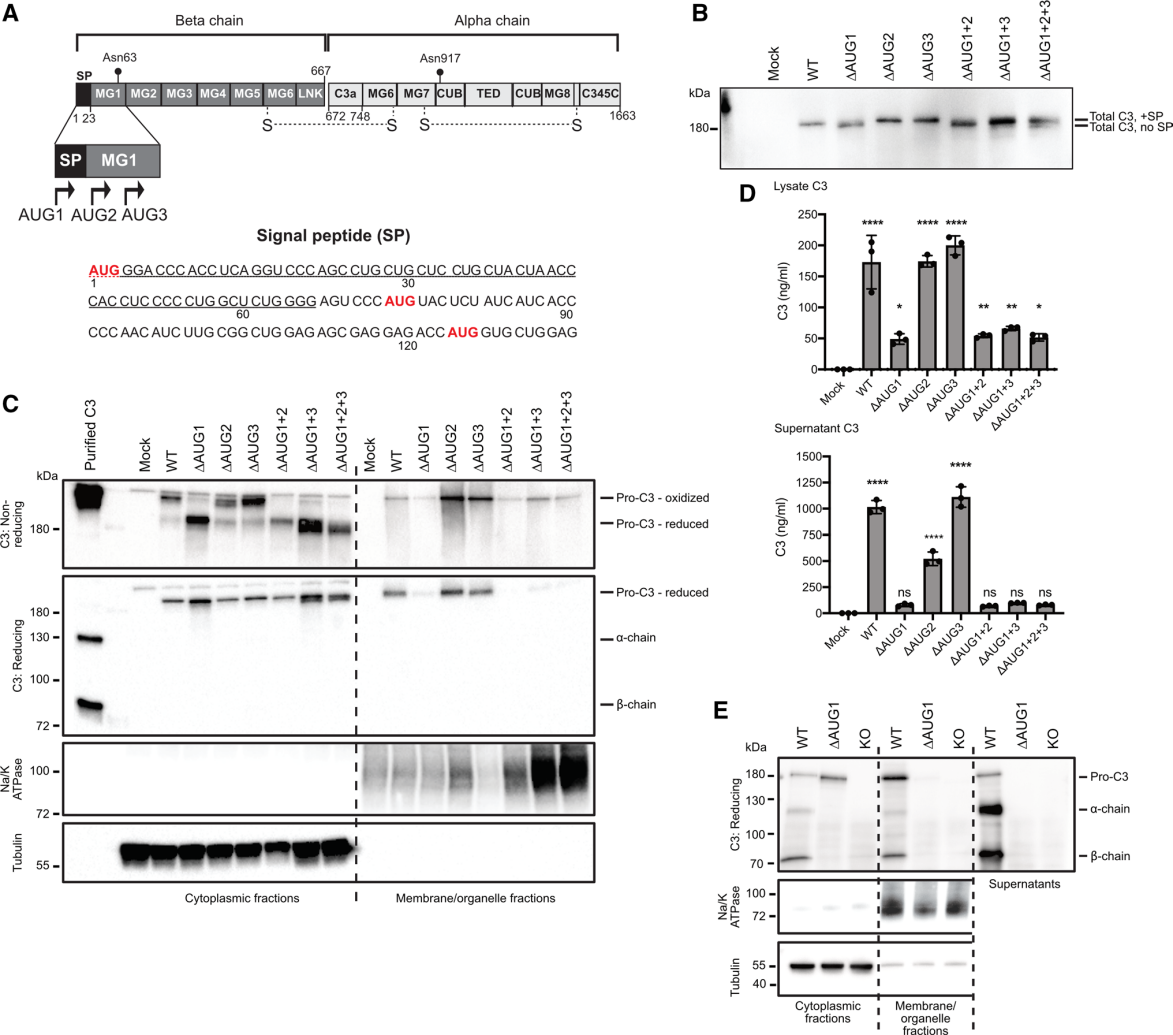
Alternative translation generates intracellular, non-secreted C3 **A** Schematic representation of C3, and 5’ sequence of C3 cDNA with potential AUG translation initiation sites (red) after the signal peptide (underlined). AUG1: canonical start site. **B** WT or AUG codon mutated C3-pcDNA3 plasmids were translated using in vitro translation and products analyzed by Western blot. **C** HEK293 cells were transfected with WT or AUG codon mutated C3 variants, and C3 expression analyzed in the soluble cytoplasmic or membrane/organelle fractions of the cells by Western blot. **D** C3 was measured by ELISA in lysates and secreted supernatants of cells in (C) (results show mean ± SD secreted C3 of 3 independent experiments carried out in duplicates). **E** Subcellular localization of C3 was investigated by Western blot under reducing conditions in A549 cells (WT, KO, or ∆AUG1 gene-edited). All blots are representative of at least *n* = 3 independent experiments. *p* > 0.05, *p < 0.05, **p < 0.01, ****p < 0.0001 compared to un-transfected cells, one-way ANOVA with Sidak’s multiple comparison

We recently proposed a potential mechanism behind generation of cytosolic C3, in the use of alternative translational start sites within native C3 mRNA, which produce a non-secreted, cytosolic C3 species due to the absence of a signal peptide [16]. Two in-frame AUG codons (denoted as AUG2, AUG3) with strong Kozak consensus sequences exist in C3 mRNA immediately after the signal peptide (Fig. 1A). The canonical (AUG1) and non-canonical (AUG2 and AUG3) start codons were mutated in a human C3 construct via site-directed mutagenesis (AUG/AUU Met/Ile substitutions) and protein translation investigated in a cell-free, in vitro translational system (Fig. 1B). Removal of the canonical AUG1 start site (∆AUG1) did not prevent C3 protein expression, but resulted in a slight gel mobility shift, consistent with an N-terminal truncation and expression of C3 isoforms lacking the signal peptide that directs the protein to the secretory pathway. Next, HEK293 cells were transfected with wild type (WT) or AUG-mutated (∆AUG) C3-pcDNA3 constructs followed by fractionation into cytosol and membrane/organelle fractions, the latter containing ER and Golgi apparatus of the secretory pathway (Fig. 1C). Removal of the AUG1 start site (and therefore signal peptide) in ∆AUG1 constructs completely prevented C3 secretion and almost completely removed C3 from the membrane/organelle fraction. Non-canonical C3 isoforms expressed from ∆AUG1 constructs were instead highly abundant in the cytosol (Fig. 1C), reflecting direct entry of the nascent protein into the cytosol during synthesis, as expected. Surprisingly, removal of all 3 AUG codons still resulted in C3 protein expression within the cytosolic fraction, showing that C3 could also be translated from non-AUG start codons [22]. Under non-reducing conditions (Fig. 1C, top panel), there was a notable migratory shift between C3 expressed from WT or ∆AUG1 constructs, which was not present under reducing conditions, suggesting different redox states of the different isoforms, consistent with translation of ∆AUG1 C3 within the reducing cytosolic environment. Notably, a band consistent with reduced cytosolic C3 was also present in HEK293 cells transfected with the WT human C3 construct, suggesting that both secreted and cytosolic C3 is produced from WT C3 cDNA, supporting the regular use of alternative translation start sites in WT cells. Presence of C3 in both the secretory pathway and cytosolic compartment was supported by enzyme-linked immunosorbent assay (ELISA) for human C3 in supernatants or total lysates of transiently transfected HEK293 cells (Fig. 1D). ∆AUG1 constructs led to detection of significant amounts of intracellular C3 (top panel), without C3 secretion (bottom panel).

These results were further supported by gene editing of A549 lung epithelial cells, which constitutively express C3 (Fig. 1E). CRISPR/Cas9 was used to produce knockout clones lacking the functional C3 gene, as well as clones with a verified frameshift mutation within the signal peptide, resulting in a truncated peptide of only 13 amino acids from the canonical start site (∆AUG1). Results from these cells, expressing C3 under the endogenous promoter in a physiological genomic context, matched those from C3 transfected HEK293 cells; only cells able to express canonical C3 from AUG1 (WT cells) had C3 in the membrane/organelle fraction and in the supernatant (Fig. 1E). Pro-C3 was found within the cytosol of WT cells and ∆AUG1 cells. Notably, only WT A549 cells expressing C3 from AUG1 had processed C3 in the cytosol; cells expressing C3 from non-canonical start sites (∆AUG1) had only pro-C3 within the cytosol.

### C3 translated from non-canonical start sites is expressed within the reducing cytosolic environment

**Fig. 2:**
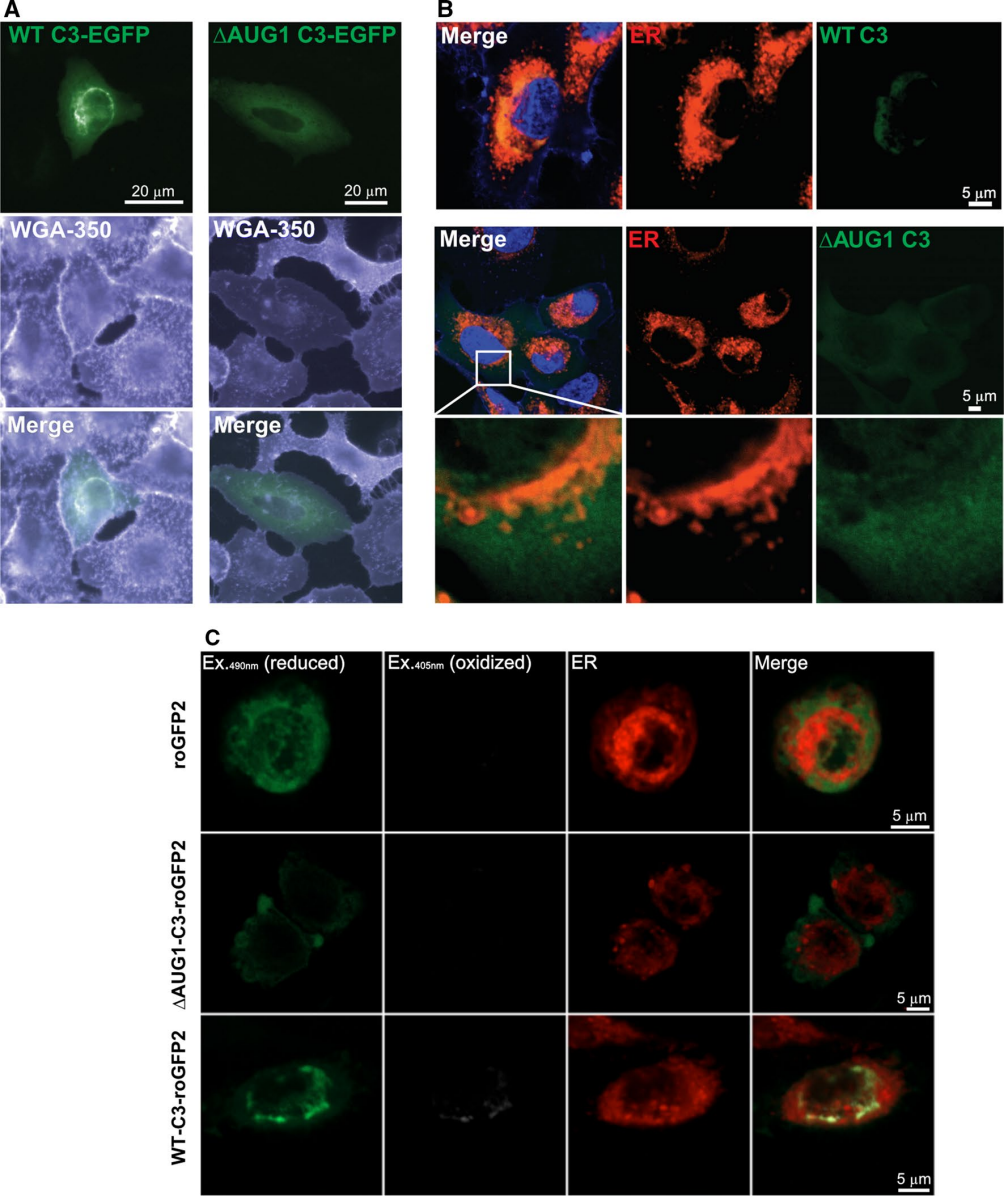
C3 expressed from non-canonical start sites is localized within the reducing environment of the cytosol. **A** WT or ∆AUG1 C3 constructs fused to C-terminal EGFP were expressed in A549 cells, co-stained with wheat germ agglutinin (WGA) and imaged by fluorescent microscopy. Both constructs led to a diffuse cytosolic stain, but only WT C3 had a concentrated peri-nuclear localization. **B** Confocal laser scanning microscopy with co-staining for ER (red) reveal that WT C3-GFP is concentrated within the ER, but that ∆AUG1 C3-GFP is excluded from the ER. **C** Use of redox-sensitive C3-roGFP2 fluorescent constructs confirms that while WT C3 is concentrated within the oxidizing ER environment, C3 expressed from non-canonical translational start sites is within the reducing cytosolic environment. All images are representative of cells imaged from at least 3 independent experiments

To confirm subcellular localization of the C3 variants, C3 knockout A549 cells were transfected with C3-EGFP fusion constructs (Fig. 2A). Intracellular WT C3 localized strongly to a perinuclear structure, most likely the early ER, but was also present diffusely throughout the cytosol. The ∆AUG1 construct, lacking a translated signal peptide required for ER entry and secretion, also lacked perinuclear localization, while diffuse cytosolic staining remained. The subcellular localization of C3-EGFP fusion proteins was confirmed by confocal microscopy (Fig. 2B), showing that WT-C3-EGFP colocalized with the ER, whereas ∆AUG1-C3-EGFP was excluded from the ER. To confirm the presence of ∆AUG1-C3 within the reducing cytosolic environment, targeted mutations were made to the constructs to introduce a disulfide bond within the GFP β-barrel, producing redox-sensitive EGFP (roGFP2) constructs, which are excited at 490 nm in reducing conditions, but additionally at 405 nm in oxidized conditions [23]. The WT, but not ∆AUG1 C3-roGFP2 protein again colocalized with the ER, and in this location was excited at 405 nm (Fig. 2C). Taken together, these results show that canonical C3 is expressed within the oxidizing intracellular secretory pathway, whereas C3 expressed from non-canonical start sites in both the WT and ∆AUG1 constructs is located within the reducing environment of the cytosol, as evidenced by cytosolic GFP fluorescence from both WT and ∆AUG1 C3-GFP constructs.

### C3 translated from non-canonical start sites is non-glycosylated, indicating ER bypass

**Fig. 3:**
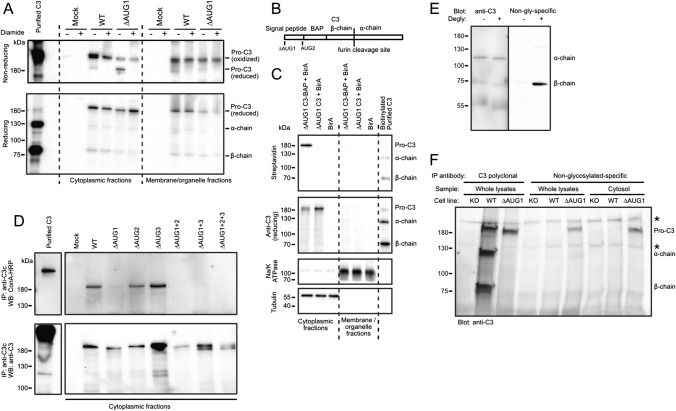
Cytosolic C3 glycosylation and redox states **A** Western blots showing that WT, cytosolic C3 is mainly in oxidized form in contrast to the ∆AUG1 variant lacking the signal peptide. HEK293 cells were transfected with WT or ∆AUG1 C3 variants. Cell lysates were separated under non-reducing and reducing conditions. Upper band represents oxidized, lower band represents reduced C3 on non-reducing SDS-PAGE. Results are representative of *n* = 2 independent experiments. **B** Scheme of the ∆AUG1 C3-BAP construct. **C** HEK293 cells were co-transfected with BirA and the canonical START site mutated ∆AUG1-BAP-C3. Biotinylated C3 was detected with streptavidin-HRP. Transfection and fractionation efficacy was analyzed by Western blotting against total C3. As loading controls, anti-human Na/K ATPase (membrane marker) and anti-human β-tubulin (cytoplasmic marker) antibodies were used. Results are representative of *n* = 4 independent experiments. **D** HEK293 cells were transfected with either the WT or START codon mutated C3 variants, the soluble cytoplasmic fraction subjected to anti-C3 IP and glycosylation investigated by Western blotting under reducing conditions using HRP-conjugated Concanavalin A. Non-reduced purified C3 was used as a control of glycosylation. Representative of *n* = 3 independent experiments. **E** Serum-purified C3 was incubated with or without deglycosylation enzymes (“degly”), and then Western blotted using polyclonal rabbit anti-C3 antibodies (left panel), or with antibodies raised against the unglycosylated human C3 β-chain N-glycosylation site peptide (right panel). Blot is representative of 3 independent repeats. **F** C3 was immunoprecipitated from lysates or cytosolic fractions of gene-edited A549 clones, using rabbit polyclonal anti-C3 antibodies (lanes 1–3), or using unglycosylated-C3-specific antibodies (lanes 4–9), and then blotted with polyclonal goat anti-C3. Results are representative of 3 independent repeats, and indicate that ∆AUG1 C3 is unglycosylated, and is found in the cytosolic fraction. Asterisks indicate nonspecific signals

In transiently transfected HEK293 cells, C3 in soluble cytoplasmic fractions migrated as two bands on non-reducing SDS-PAGE, of which the lower band is more pronounced in case of the ∆AUG1 variants (Fig. 1C). Since on reducing SDS-PAGE only a single, 180 kDa isoform of all variants could be detected (Fig. 1C), we hypothesized that the two isoforms observed under non-reducing conditions in the cytosol are the oxidized and reduced forms of the protein. After diamide treatment, which oxidizes free thiol groups [24], cytosolic C3 runs as a single species on non-reducing SDS-PAGE (Fig. 3A), confirming that the lower band is indeed a reduced form of the protein, consistent with expression within the reducing environment of the cytosol, bypassing the ER where disulfide bridges are formed. To again verify the cytosolic location of ∆AUG1 C3, a ∆AUG1 C3 construct was produced with an introduced biotin acceptor peptide (BAP [25]) inserted directly after AUG2 (Fig. 3B), preventing C3 secretion, but meaning that any protein product from a non-canonical start site at or upstream of AUG2 will be a substrate for the cytosolic BirA enzyme, which was co-expressed in the same cells (Fig. 3C). C3 was indeed biotinylated when BirA was co-expressed in the same cells, confirming that C3 and BirA are in the same compartment and therefore that C3 translated from a downstream non-canonical start site is cytosolic.

Canonical C3 is glycosylated at Asn63 and Asn917 in the ER lumen. To assess the glycosylation status of C3 variants, C3 was immunoprecipitated from soluble cytoplasmic fractions of transfected HEK293 cells and tested for Concanavalin A binding (Fig. 3D). Both WT and signal peptide-containing C3 variants (∆AUG2 and ∆AUG3) reacted strongly with the lectin, indicating that the proteins are glycosylated (Fig. 3D) and that some glycosylated C3 is translocated from the site of glycosylation into the cytosol. In contrast, all four ∆AUG1 C3 variants lacking the signal peptide are not glycosylated (∆AUG1, ∆AUG1 + 2, ∆AUG1 + 3, ∆AUG1 + 2 + 3), verifying bypass of the ER.

To confirm this result, we raised polyclonal antibodies specific for unglycosylated C3, which only recognized the N-glycosylation peptide motif of serum-purified C3 β-chain after incubation with deglycosylation enzymes (Fig. 3E). In comparison to polyclonal rabbit anti-C3, which immunoprecipitated C3 from both WT and ∆AUG1 A549 cells, the unglycosylated-specific antibody only immunoprecipitated detectable levels of C3 from ∆AUG1 cells (Fig. 3F), indicating that this form is not glycosylated. Together, these results confirm that C3 translated from non-canonical start sites is reduced and unglycosylated, consistent with its site of translation in the cytosol.

### Canonical C3 is also retrotranslocated to the cytosol

**Fig. 4:**
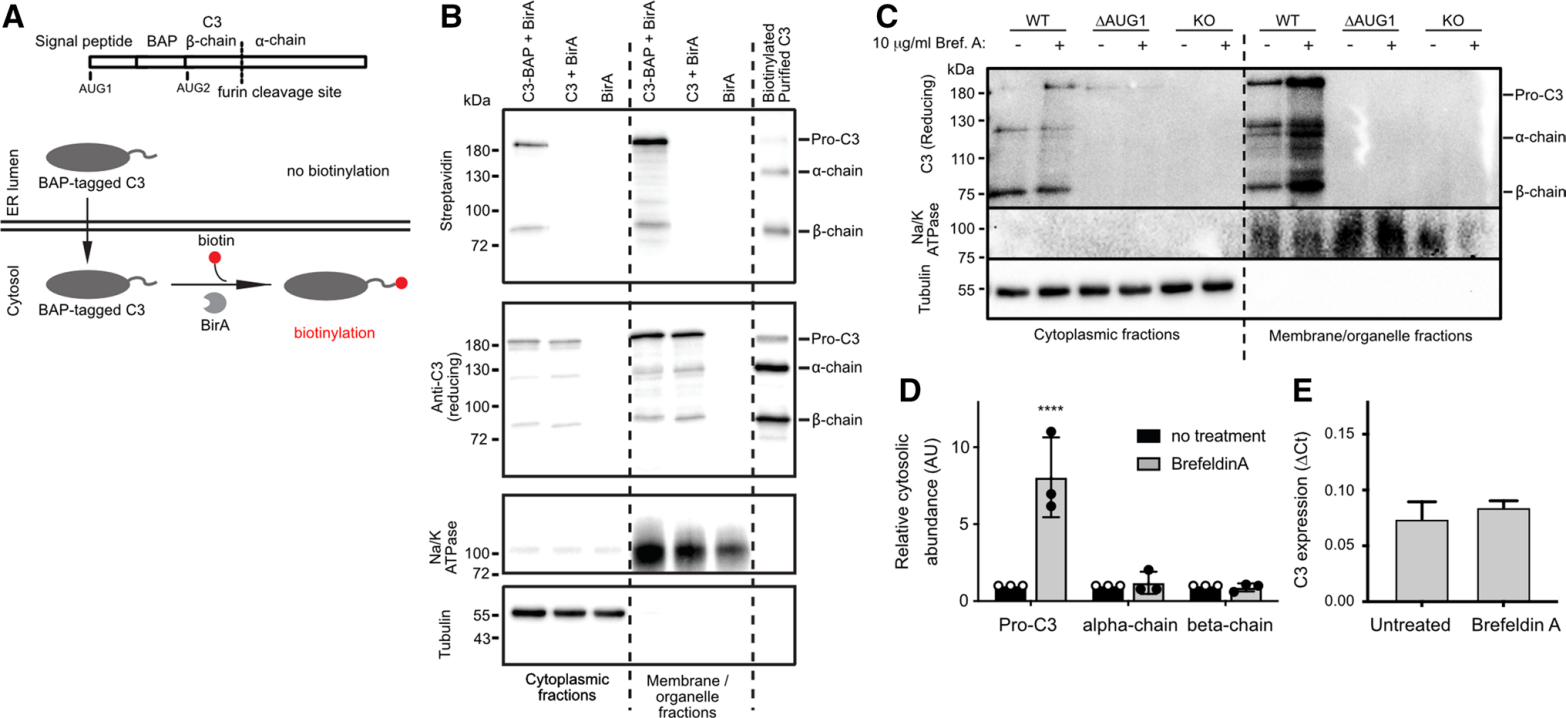
Retrotranslocation also contributes to maintenance of cytosolic C3 level. **A** Scheme of in vitro biotinylation of BAP-tagged C3. Only retrotranslocated BAP-C3 will be biotinylated by cytosolic BirA. **B** HEK293 cells were co-transfected with BirA and canonical BAP-C3. Biotinylated C3 in the cytosol–reflecting retrotranslocated C3–was detected using streptavidin-HRP. Results are representative of *n* = 3 independent experiments. **C** WT or ∆AUG1 A549 cells were treated with 10 µg/ml Brefeldin A for 4 h to inhibit C3 secretion. After fractionation, samples were separated on reducing SDS-PAGE with subsequent Western blotting using anti-C3 Ab. The relative abundance of pro-C3, C3-α- and C3-β in the cytosol of WT cells were quantified using ImageLab software, shown in (**D**). **E** qPCR for C3 expression in A549 cells after Brefeldin A treatment. Results are representative of *n* = 3 independent experiments (two-way ANOVA with Dunnett’s multiple comparison: ****p < 0.0001)

Presence of processed (Fig. 1E), oxidised (Fig. 3A), and glycosylated C3 (Fig. 3D) in the cytosolic fractions of cells expressing canonical C3 also suggests retrotranslocation of C3 from the Golgi/ER lumen into the cytosol. We again used C3-BAP constructs co-expressed with BirA to investigate retrotranslocation[26]. This time we inserted the BAP directly after the C3 signal peptide cleavage site, but upstream of AUG2 (Fig. 4A), meaning that canonical C3 becomes biotinylated only if retrotranslocated to the cytosolic site of the BirA enzyme. In contrast to ∆AUG1-BAP-C3 (Fig. 3C), biotinylated processed C3 β-chain was found in the cytosol (Fig. 4B), demonstrating that retrotranslocation of canonical C3 occurs after furin-dependent processing. Biotinylated C3 also associated with the membrane-organelle fraction, indicating that retrotranslocated C3 may interact with intracellular organelles or membrane structures.

The presence of processed C3 within cytosol fractions could indicate the inclusion within this fraction of small vesicles containing secretory C3. To test this, A549 cells were treated with Brefeldin A, which inhibits transport of proteins from ER to Golgi complex (Fig. 4C). Brefeldin A prevented C3 secretion and caused accumulation of C3 within the membrane/organelle compartment, indicating successful inhibition of the secretory pathway. In comparison, levels of C3 alpha- and beta-chain in cytosolic fractions were not affected (Fig. 4D), indicating that these are not canonical C3 within the secretory pathway. Furthermore, cytosolic levels of pro-C3 increased, possibly due to increased retrotranslocation caused by Brefeldin A-induced ER stress. These changes were not due to altered C3 gene transcription, as measured by qPCR (Fig. 4E).

### Cytosolic C3 is turned over by the ubiquitin–proteasome system

**Fig. 5:**
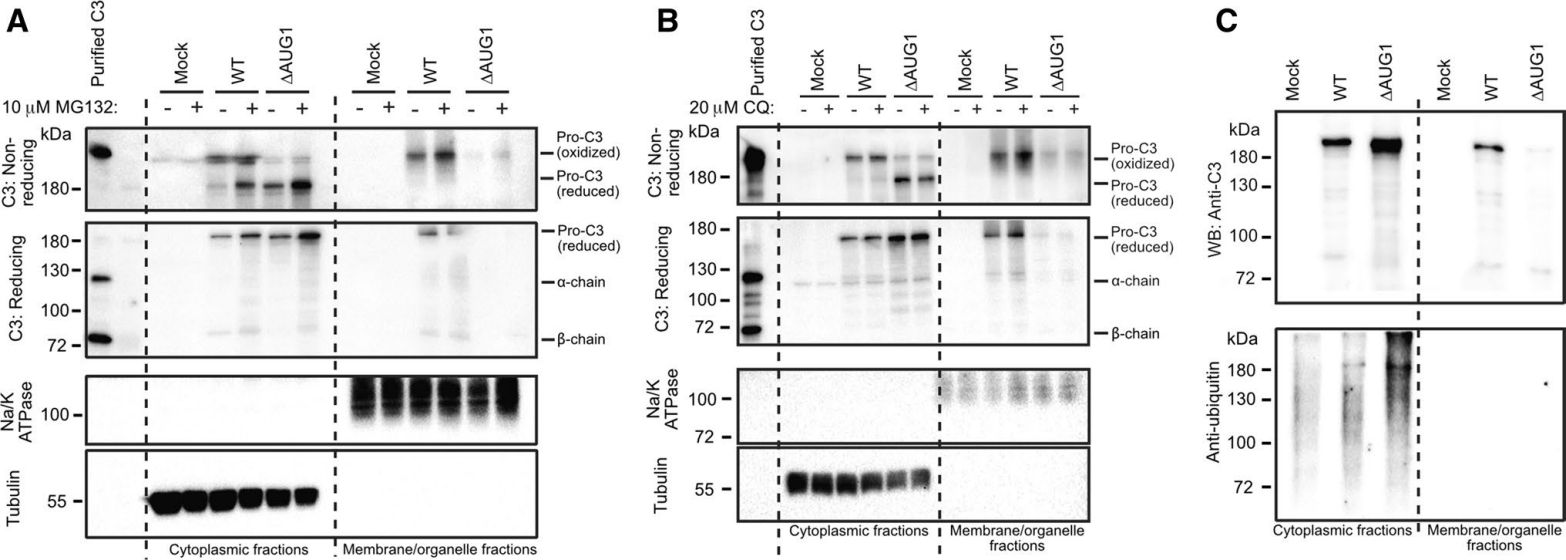
Cytosolic C3 is turned over by the ubiquitin–proteasome system **A** HEK293 cells were transfected with either WT or ∆AUG1 C3 and treated with 10 µM MG-132 proteasome inhibitor. Cell lysates were fractionated and run on non-reducing (top panel) or reducing (second panel) SDS-PAGE followed by Western blotting. Data shown are representative of *n* = 3 independent experiments. **B** HEK293 cells were transfected with either the WT or ∆AUG1 C3 and treated with 20 µM chloroquine (CQ) to inhibit autophagy. Expression of C3 was investigated on non-reducing and reducing SDS-PAGE by Western blot and are representative of *n* = 2 experiments. **C** WT or canonical START codon mutated C3 were expressed by HEK293 cells, immunoprecipitated and ubiquitinylation assessed by Western blotting using the anti-ubiquitin Ab, P4D1. Data are representative of *n* = 2 independent analyses

To investigate cytosolic C3 turnover by the ubiquitin–proteasome or autophagy degradation systems, transfected HEK293 cells were treated with the proteasome inhibitor MG-132 (Fig. 5A) or with autophagy inhibitor chloroquine (Fig. 5B). MG-132 treatment increased levels of cytosolic C3 (Fig. 5A), while C3 in the membrane fraction was unaltered. The effect of proteasome inhibition was most pronounced on the reduced, ie. non-canonically translated cytosolic C3 (lower band), leaving the retrotranslocated oxidized C3 fraction unaltered (Fig. 5A, upper band in non-reducing conditions). In comparison, chloroquine treatment did not affect intracellular C3 levels (Fig. 5B). These results indicate that cytosolic non-canonically translated C3 is turned over by the proteasome. In support of this, C3 immunoprecipitated from the cytosol was ubiquitinylated, in contrast to C3 from the membrane/organelle secretory pathway (Fig. 5C).

### Cytosolic C3 functions in intracellular pathogen detection and opsonization

**Fig. 6:**
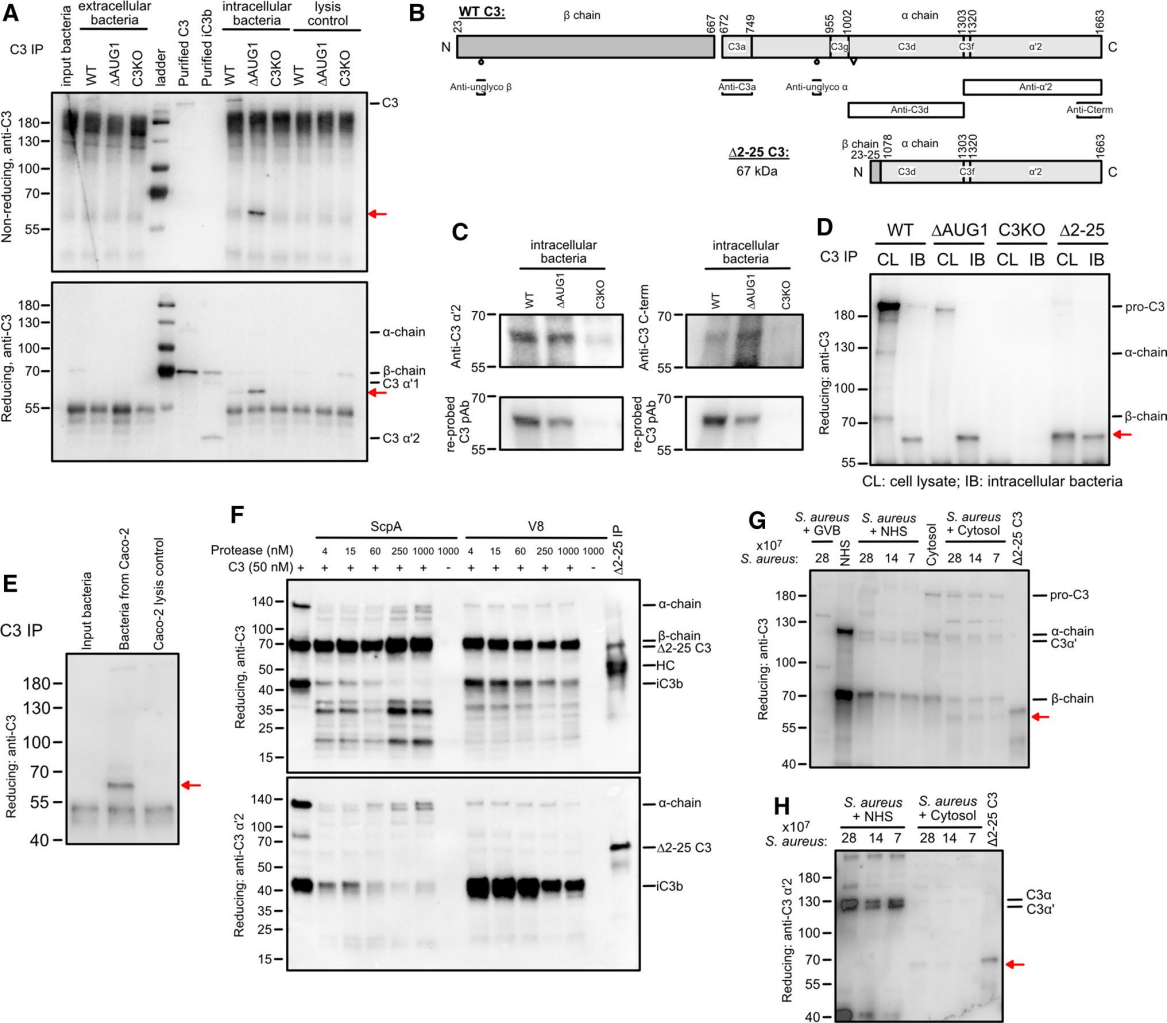
Cytosolic C3 from alternative translational start sites opsonizes cytoinvasive *Staphylococcus aureus.* **A** WT, ∆AUG1 and C3KO gene-edited A549 cells were infected with log-phase *S. aureus* for 1 h, extracellular bacteria removed and collected, and gentamicin added to kill remaining un-internalized bacteria. After 18 h, intracellular bacteria were recovered by lysing A549 cells. C3 fragments deposited on these bacteria were detached by incubation in PBS containing 50 mM methylamine, and recovered by immunoprecipitation, followed by Western blot under reducing (top panel) or non-reducing (bottom panel) conditions with anti-C3 polyclonal antibody. Lysis control, uninfected cells. **B** Scheme of mature processed C3, and epitopes detected by various tested antibodies. A comparison of the truncated ∆2–25 C3 product is also shown. Amino acid numbering is based on pre-pro-C3 that includes the 22 amino acid signal peptide that is removed in the ER. C3 glycosylation sites and thioester group are annotated as circles and triangle, respectively. **C** Intracellular bacteria samples were concentrated by TCA precipitation, followed by Western blot with tested monoclonals anti-α’2 and anti C-term (upper panels), which recognize epitopes indicated in 6B. Membranes were stripped and re-probed using polyclonal anti-C3 to confirm identity of the fragments (bottom panels). **D** Cell lysate (CL) of WT, ∆AUG1, C3KO and ∆2–25 A549 cells, and intracellular bacteria (IB) samples from the same cells were immunoprecipitated for C3, followed by Western blot with a polyclonal anti-C3 antibody. **E** C3 fragments from *S. aureus* infecting Caco-2 cells were also collected by immunoprecipitation, followed by Western blot with an anti-C3 polyclonal antibody. **F** Purified C3 was incubated with increasing concentrations of the Staphylococcal proteases ScpA and V8 at 37 °C for 1 h. Samples were separated on SDS-PAGE with subsequent Western blot using polyclonal (upper panel) and anti-α’2 (lower panel) C3 antibodies. The ∆2–25 C3 fragment, obtained by immunoprecipitation of C3 from ∆2–25 A549 cells was loaded as a reference (final lane). **G** Log-phase *S. aureus* were incubated with NHS or hepatocyte cytosolic fraction. C3 fragments deposited on bacteria were blotted with anti-C3 polyclonal antibody. Samples of NHS and cytosol fraction were loaded in parallel together with the ∆2–25 C3 fragment immunoprecipitated from ∆2–25 A549 cells. **H** Samples shown in panel (G) were also blotted using the anti-α’2 C3 antibody recognizing the C-terminus of C3 alpha-chain. All blots are representative of at least 3 independent repeats

C3-opsonized cytoinvasive pathogens can trigger cell innate immunity and xenophagy upon cell entry [17, 27]. To investigate whether cytosolic C3 can also detect intracellular pathogens, we infected gene-edited human lung epithelial A549 cells with *S. aureus*, which invades into the cytosol of these cells [28]. Intracellular bacteria retrieved from WT and ∆AUG1 A549 cell lysates, but not C3-KO A549 cells, were associated with a ~ 65 kDa fragment of C3 as detected by polyclonal anti-C3 antibody (Fig. 6A). We used a panel of antibodies to map this fragment (Fig. 6B). It was recognized by monoclonal antibodies against the C3 α’2 fragment and C-terminus of the α-chain (Fig. 6C), but not by antibodies recognizing N-glycosylation epitopes, nor monoclonals against C3a or C3dg regions (Fig. S1). This indicates that a C-terminal 65 kDa fragment of the α-chain of cytosolic C3 is deposited on intracellular bacteria.

To verify this, we produced gene-edited A549 clones expressing this truncated region of C3, by using CRISPR/Cas9 to excise exons 2–25 of the human C3 gene (∆2–25) encoding the majority of the β-chain and the N-terminal portion of the α-chain, including the thioester group (Fig. 6B, S2A). The resultant spliced mRNA did not contain a frameshift. Western blotting showed that the resultant truncated C3 protein of roughly 65 kDa is expressed within the membrane/organelle fraction as well as in the cytosol (Fig. S2B), but was not secreted. Infection of ∆2–25 A549 cells expressing this truncated C-terminal C3 fragment also led to bacterial deposition of a C3 fragment similar in size to that deposited by WT or ∆AUG1 cells, (Fig. 6D), confirming that this C-terminal domain of intracellularly expressed C3 α-chain interacts with *S. aureus*.

*Staphylococcus aureus* is an opportunistic pathogen, which infects a variety of epithelia: pulmonary, oral, nasal, gastrointestinal, epidermal, and urogenital [28, 29]. We therefore investigated cytosolic C3 deposition on *S. aureus* in gut epithelial cells. We found that the same 65 kDa fragment was deposited on *S. aureus* upon infection of Caco-2 gut epithelial cells (Fig. 6E), suggesting that this mechanism is not restricted to lung epithelial cells.

Several *S. aureus* proteases are known to cleave the C3 α-chain [30], including cysteine protease staphylopain A (ScpA) and serine protease V8. Purified C3 was incubated with ScpA and V8, but no 65 kDa products were detected by anti-C3 α’2 antibody (Fig. 6F), indicating that the C-terminal C3 product associating with intracellular bacteria is not produced by these two staphylococcal proteases. We then asked whether cytosolic-specific factors are required for generation of the 65 kDa C3 fragment. Incubating *S. aureus* with human serum lead to C3 deposition, with α- and β-chains visible by Western blot, and cleavage of C3 into C3b, identifiable by the slightly smaller α'-chain (Fig. 6G). However, incubation of *S. aureus* with purified human hepatocyte cytosol fractions also led to a ~ 65 kDa fragment product highly similar to that deposited during *S. aureus* infection of A549 and Caco-2 cells, detectable by both polyclonal anti-C3 (Fig. 6G) and anti-C3 α’2 antibodies (Fig. 6H). This fragment was absent when *S. aureus* was incubated with normal human serum (NHS), indicating that production of the 65 kDa C-terminal C3 fragment is dependent on cytosolic components.

### Cytosolic C3 impacts cytosolic bacteria virulence

**Fig. 7:**
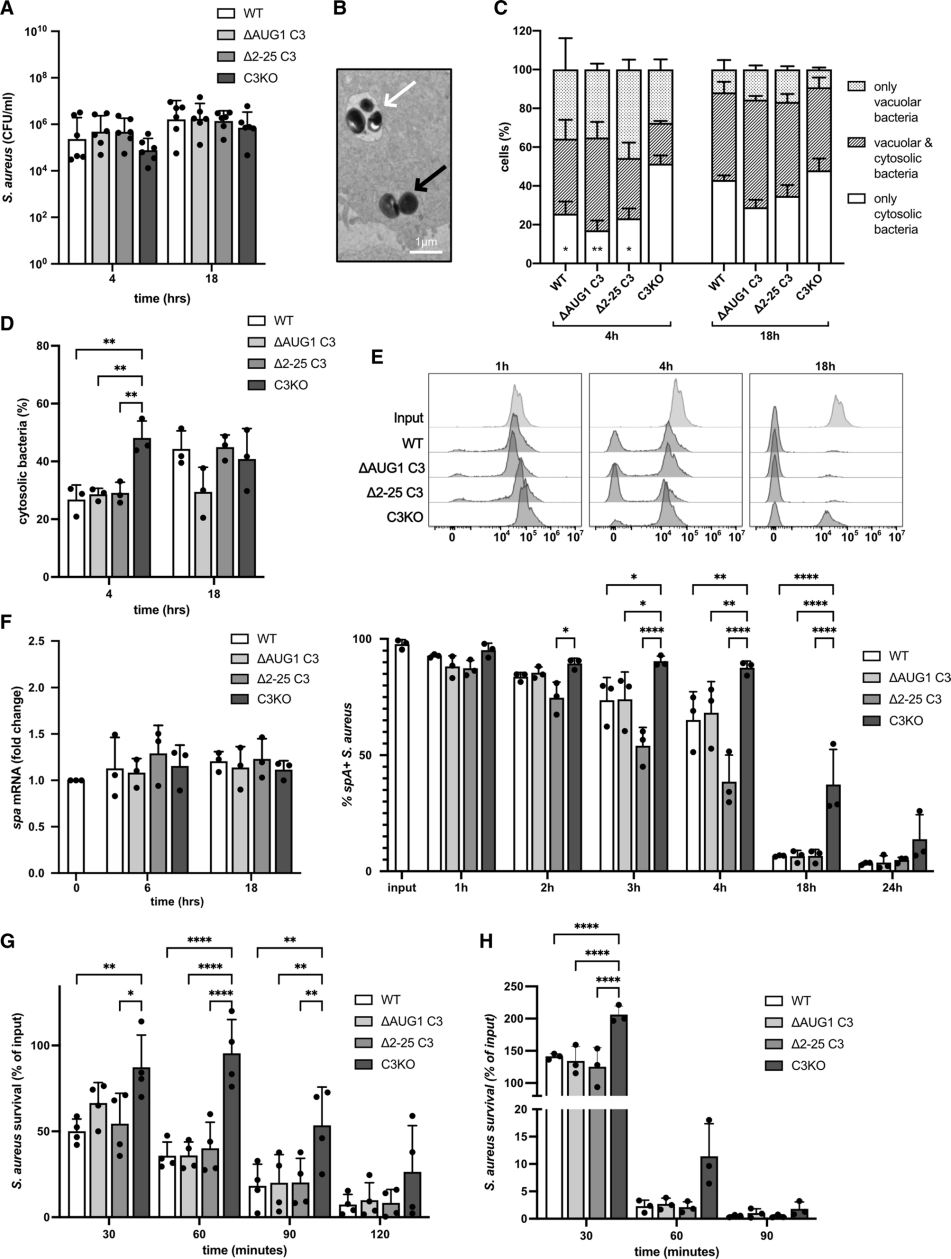
Cytosolic C3 affects cytosolic bacteria. **A** WT and gene-edited A549 cells were infected with log-phase *S. aureus* for 1 h, then gentamicin added to kill extracellular bacteria. After 4 or 18 h incubation, intracellular bacteria were recovered by cell lysis. Bacteria were plated and CFU counted after 24 h at 37 °C. **B** WT, ∆AUG1, ∆2–25, and C3KO A549 cells were infected with log-phase *S. aureus* for 4 h or 18 h. Cells were collected, fixed and analyzed by electron microscopy. Cytosolic (black arrow) and vacuolar bacteria (white arrow) were clearly distinguishable. **C** The percentage of infected cells harboring only vacuolar bacteria, only cytosolic bacteria, or both was assessed. Statistical difference was found in cytosolic bacteria in C3KO cells compared to WT, ∆AUG1 and ∆2–25 cells at 4 h, other comparisons were not significant. **D** The percentage of cytosolic bacteria among all invading bacteria was also calculated. **E** Intracellular bacteria were collected by lysis of WT, ∆AUG1, ∆2–25, and C3KO A549 cells at 1, 2, 3, 4, 18 and 24 h post-infection. Bacteria were gated using fluorescent vancomycin-BOPIDY, and SpA expression detected by flow cytometry. Shown are representative histograms (upper panel) and percentage of SpA + bacteria (lower panel). **F** Intracellular bacteria were collected by lysis of WT, ∆AUG1, ∆2–25, and C3KO A549 cells at 6 and 18 h post-infection, followed by semi-quantitative RT-PCR for estimation expression levels of *spa* mRNA. **G** Intracellular bacteria extracted from WT, ∆AUG1, ∆2–25, and C3KO A549 cells were added to fresh human blood, and bacteria survival after shown timepoints was assessed by counting CFU on blood agar plates. Survival was calculated as percentage of input for each A549 cell type from each individual experiment. **H** Intracellular bacteria extracted from WT, ∆AUG1, ∆2–25, and C3KO A549 cells were added to differentiated HL60 cells and bacteria survival was assessed by counting CFU on blood agar plates. All results represent mean ± SD of 3–6 independent repeats. *p < 0.05, **p < 0.01, ***p < 0.001, ****p < 0.0001. A, One-way ANOVA with Dunnett’s multiple comparison post-test, C-H Two-way ANOVA with Dunnett's multiple comparison post-test

C3 opsonization can induce intracellular bacterial killing by autophagy, restricting bacterial survival within colon epithelial cells [17]. We therefore assessed whether cytosolic C3 impacts *S. aureus* survival within lung epithelial cells. Similar numbers of intracellular bacteria were collected from infected C3-KO or WT A549 cells, demonstrating that cytosolic C3 does not alter intracellular *S. aureus* survival (Fig. 7A). *S. aureus* is internalized by endocytic uptake, before escaping into the cytosol [31], where they could encounter cytosolic C3. Using electron microscopy, we assessed the intracellular localization of *S. aureus* in A549 cells at 4 and 18 h post-infection: cytosolic or vacuolar bacteria are clearly distinguishable (Fig. 7B). Between 4 and 18 h post-infection, proportions of WT cells harboring cytosolic bacteria increased, and cells harboring only vacuolar bacteria decreased, illustrating the escape of *S. aureus* from vacuoles into the cytosol (Fig. 7C). The same result was seen quantifying the percentage of cytosolic bacteria, which increases in WT cells between 4 and 18 h (Fig. 7D). Interestingly, a significantly larger proportion of C3-KO cells harbor only cytosolic bacteria at 4 h post-infection compared to C3-expressing cells (WT, ∆AUG1, and ∆2–25) (Fig. 7C) and contain significantly more cytosolic bacteria (Fig. 7D). This suggests that cellular C3 expression delays the escape of *S. aureus* into the cytosol.

*Staphylococcus aureus* gene expression and phenotype change profoundly upon internalization into human cells [32, 33]. We used flow cytometry to assess *S. aureus* expression of virulence factor Staphyloccus protein A (SpA) during A549 cell infection. Log-phase planktonic bacteria expressed high levels of surface SpA, which was quickly downregulated upon internalization into WT cells (Fig. 7E), as described previously [32]. Similar SpA downregulation was also observed within ∆AUG1 and ∆2–25 A549 cells, but was significantly delayed in C3-KO A549 cells. This suggests that in the absence of cytosolic C3, the intracellular environment provokes a different response in invasive bacteria. Interestingly, levels of SpA mRNA were similar in all intracellular bacteria, indicating that levels of surface SpA was controlled by post-transcriptional regulation (Fig. 7F). Several other virulence factors were also assessed: although mRNA levels increased in intracellular bacteria compared to input bacteria, there was no difference between intracellular bacteria from C3-expressing or C3-KO A549 cells (Fig. S3). These results suggest that the intracellular environment faced by *S. aureus* is altered in C3-KO cells, impacting expression of selected virulence factors, but not intracellular survival.

We next asked whether intracellular deposition of cytosolic C3 alters targeting of *S. aureus* by phagocytes upon release from epithelial cells. Intracellular bacteria collected from WT, ∆AUG1, ∆2–25, and C3-KO A549 cells were incubated with whole human blood. Bacteria collected from C3-KO A549 cells displayed increased survival compared to those from all three C3-expressing A549 cells (WT, ∆AUG1, ∆2–25) (Fig. 7G), consistent with the hypothesis that deposition of the 65 kDa cytosolic C3 fragment on *S. aureus* increases killing by phagocytosis. To confirm this in a simplified system, the neutrophil-like phagocytic cell line HL60 was used, which are capable of complement receptor-dependent uptake and killing [34]. In absence of human serum, *S. aureus* collected from C3-KO A549 cells experienced less HL60-induced killing than bacteria from all C3-expressing cells (Fig. 7H), suggesting that the increased whole-blood survival of bacteria from C3-KO cells is also due to decreased phagocytosis dependent on intracellular cytosolic C3 deposition.

## Discussion

**Fig. 8:**
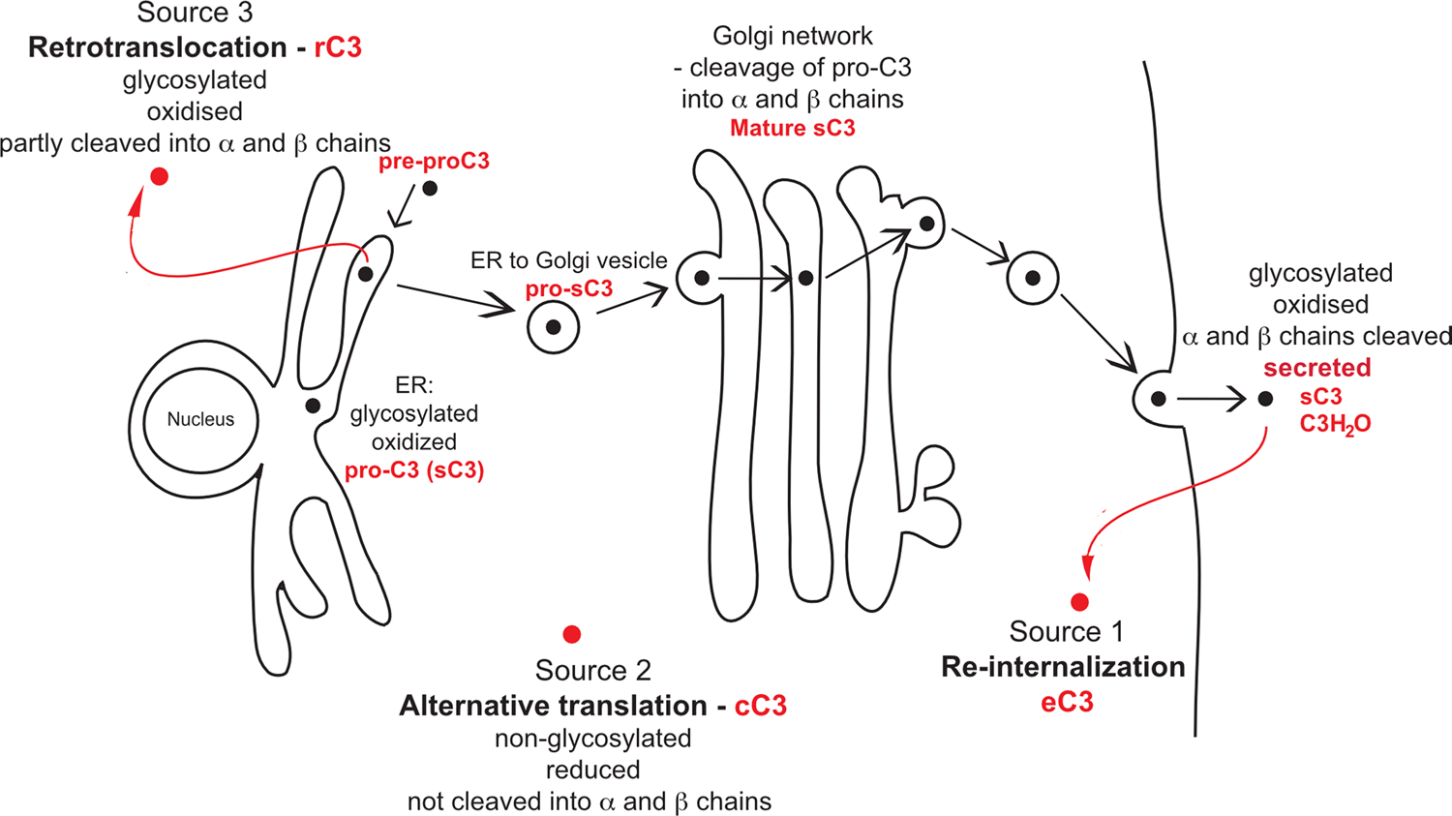
Model for the origin of intracellular, cytosolic C3. Serum C3 is synthesized on ER-associated ribosomes as pre-proC3 with a signal peptide, which targets the protein to the ER. During ER entry, the signal peptide is cleaved, generating pro-C3, which becomes glycosylated in the ER lumen at Asn63 and Asn917. After quality control, proC3 is further transported to the Golgi, cleaved into α- and β-chains, producing a mature C3, which is secreted from the cell (“sC3”). In contrast to the secreted form, cytosolic C3 may have three different sources. Source 1 is intracellular C3 originating by uptake from the extracellular space, i.e. serum or locally produced C3, which is internalized by the cells (“eC3) [21]. eC3 species is present mainly in cells, which are surrounded or migrated through the blood and is identical with serum C3. Next to the extracellular origin, many cells express C3 endogenously. In the latter case, C3 is present also in the cytosol, and this form is either generated via alternative translation from AUG codons following signal peptide coding sequences (“cC3”, Source 2) or via ERAD-associated retrotranslocation (“rC3”, Source 3), producing C3 species which are non-glycosylated, reduced and partially degraded by the ubiquitin–proteasome system. *cC3* cytosolic C3, *eC3* endosomal C3, *rC3* retrotranslocated C3, *sC3* secreted C3

Besides its classical roles within innate and adaptive immunity, complement is emerging as a key mediator of basic cellular processes [4]. Importantly, C3, the central complement protein, is expressed in many cell types, and orchestrates complex physiological processes in autocrine as well as intracellular manners [3]. Despite the growing interest in intracellular C3, its generation and subcellular localisation is not completely understood. In this study we have characterized C3 within the cytosol itself, the gel-like fluid filling the cell volume, which together with the organelles suspended within it, makes up the cytoplasm. We have described two novel mechanisms, alternative translation and retrotranslocation, by which C3 could enter the cytosol (Fig. 8), based on the presence of both processed C3, as well as non-glycosylated pro-C3, within cytosolic cell fractions. Further, we show that a fragment of cytosolic C3 opsonizes invasive *S. aureus*, leading to enhanced phagocytosis after release of bacteria into the extracellular environment.

The use of alternative translational start sites allows a large number of eukaryotic proteins to be produced from the surprisingly few number of genes due found in the human genome [35], and is a recognized mechanism for controlling production of alternative protein isoforms with altered function or subcellular localization. This has been demonstrated for multiple human proteins [36–38], including important components of innate and adaptive immunity [39–41]. Here we show that alternative translational start sites in the same mRNA molecule can be exploited to generate both secreted (sC3), and non-secreted cytosolic forms (cC3) of C3, without use of multiple splice variants. The isoforms generated by this mechanism localize to distinct intracellular compartments and may mediate different functions. Human C3 contains two in-frame AUG codons downstream from the signal coding sequence with strong Kozak consensus where translation may initiate (Fig. 1A). Interestingly, mutation of both of these AUG codons did not lead to complete inhibition of translation (Fig. 1B, C), indicating the presence of other, non-AUG translation start sites around the signal peptide. Although it was previously thought that translation initiates only at AUG start codons, non-AUG start codons are also used at a high frequency [22]. This can be caused by ribosomal leakage, when ribosomes 'skip' the canonical AUG start codon and continue downstream until they recognize another Kozak-like sequence and/or a stable mRNA hairpin secondary structure [35, 42]. VEGF [43] and ostopontin [44] are further examples that can be translated from alternative non-AUG codons, generating both secreted and intracellular isoforms. In the case of VEGF, the well-known secreted regulator of vascularization, intracellular functions of intracellular isoforms in regulation of cell growth, survival and metabolism have also been identified, in what has been termed as “intracrine” functions [45]. The same mechanism may also operate in the case of human C3. The canonical, secreted isoform dominates over alternatively translated C3 probably due to the stronger Kozak consensus and retention of the ribosomes on the first available AUG codon. Nevertheless, tissue- and cell-type specific factors, as well as cellular stress [46], could result in the usage of alternative translation initiation sites, up-regulating non-canonical isoforms. For C3, this could provide protective or homeostatic roles in pathogen defense [27], autophagic relief of stress [47], and defense against cytokine-induced apoptosis [48] or oxidative stress [15]. The use of alternative translational start sites therefore allows both a diversity and appropriate responsive control of protein functions. Ongoing studies are necessary to understand and verify the mechanisms behind regulation of subcellular C3 expression and localization, and to identify the exact translational initiation sites and mechanisms within C3 mRNA.

As well as the reduced and non-glycosylated isoform of C3 originating from expression directly within the cytosol, a portion of cytosolic C3 is glycosylated and oxidized, indicating its ER origin. Retrotranslocation allows ER proteins access to the cytosol, where they can have additional functions [49]. Similarly to C3, clusterin can also be retrotranslocated from ER to cytosol, where it also exerts distinct functions [50]. Previously, it was assumed that proteins must be unfolded before transport through narrow pores from ER to cytosol, but it has been demonstrated that structure and disulfides can be retained during retrotranslocation [51]. The presence of various intracellular C3 species raises the question as to which mechanisms are responsible for the switch between the distinct isoforms, and whether they exert different functions, which both are likely dependent on the cell type.

Finally, we have identified functions for the cytosolic isoform of C3. We and others have previously shown that C3 interacts with ATG16L1 in regulation of autophagy [16, 17], suggesting that C3 functions both as an intracellular as well as extracellular opsonin. Further, C3 brought into the cell on the surface of complement-opsonized cytoinvasive pathogens can trigger cell autonomous immunity [27]. We now show that endogenous cytosolic C3, present in a lung epithelial cell line, is capable of depositing on cytoinvasive bacteria in the absence of extracellular C3. *S. aureus* invade and replicate in epithelial cells, where they can persist for prolonged periods of time [52], before inducing apoptosis and being released to spread infection. The interaction of this bacteria with intracellular innate immune detection pathways is therefore important in control and clearance of infection. While the deposition of C3 fragments was absent in C3KO cells, an identical C3 fragment was deposited in both WT and ∆AUG1 cells, the latter of which only express C3 within the cytosol, and lack secreted C3. We mapped this deposited fragment to a roughly 65 kDa portion of the C-terminal region of the C3 α-chain, a fragment that does not correspond to cleavage products of canonical complement activation pathways. This fragment was not produced by incubation of purified C3 with C3-cleaving staphylococcal enzymes, or by incubation of bacteria with human serum, but required the additional presence of human cytosolic factors. Gene-edited ∆2–25 A549 cells, where exons 2–25 of C3 were excised from the genome, express a similar 65 kDa C3 fragment, which was also deposited on to intracellular *S. aureus*. Interestingly, we have identified a predicted ATG16L1-interaction motif [53] within this region of C3 [18]. Compared to C3KO A549 cells, *S. aureus* infecting other cell types expressing the 65 kDa C3 fragment showed slower vacuolar escape, and a slower downregulation of expression of SpA, a secreted and surface-bound immunoglobulin-binding protein critical for opsonophagocytosis evasion [54] that is downregulated upon bacterial internalization. The deposited region of C3 does not contain the thioester group of C3, but contains a complement receptor 2 (CR2) binding motif exposed on the C3d surface [55]. Perhaps as a result of this, bacteria released from C3-expressing A549 cells, including ∆AUG1 and ∆2–25 cells, were more susceptible to phagocytosis and killing by CR2-expressing HL60 cells and in whole blood, when compared to C3KO A549 cells. As a gram-positive bacteria, *S. aureus* is resistant to direct complement-mediated lysis, and phagocytosis is therefore the main mechanism of killing in whole blood. The intracellular opsonization of bacteria with C3, as demonstrated here in A549 lung epithelial cells as well as Caco-2 gut epithelial cells, may be of increased importance in epithelium, which faces direct exposure to potential pathogens in an environment where serum proteins, including C3, are present at lower levels than found in blood or tissue fluids [56]. This strongly implies a potential function of intracellular, cytosolic C3 in pathogen detection in C3-expressing epithelial cells at the host-environment interface. Here, the release of intracellular C3-coated pathogens might lead to their early detection and elimination by mucosal-associated lymphoid tissue immune phagocytes, independent of serum components. The form of C3 that we find deposited on intracellular *S. aureus* in our experimental system is a truncated C-terminal fragment of the α-chain, which would necessitate cleavage of cytosolic C3 by as-yet unidentified factors. Further work is now required to develop these proof-of principle results in infectious models and in vivo systems, for physiological verification. Intracellular C3 has already been shown to be able to trigger cell autonomous immunity [27] and target intracellular destruction of pathogens [17], and this together with our current findings implies that the well-known role of complement and C3 in extracellular innate immunity, is also paralleled by a function of cytosolic C3 within the intracellular environment, in pathogen detection and defense.

## Materials and methods

### Cells and media

HEK293, A549 and Caco-2 cells were maintained in DMEM (Gibco) with 10% heat inactivated fetal bovine serum (FBS, Gibco). HL60 cells were grown in RPMI 1640 (Gibco), 10% FBS. All cells were tested regularly for mycoplasma contamination (Eurofins Genomics) and cultured at 37 °C in 5% CO_2._

### Creation of gene-edited A549 cells

Guide RNA sequences targeting the canonical AUG and second AUG codons of the human C3 gene were cloned into the pX459 plasmid [57], a gift from Feng Zhang (Addgene plasmid #62,988). For targeted mutagenesis of the second AUG codon by homology directed repair (HDR), a single stranded DNA oligonucleotide incorporating a AUG/CTG mutation of the AUG site and silent mutation of the upstream PAM site was co-transfected with the guide RNA-containing pX459 plasmid. After transfection, individual clones were screened for C3 secretion and by amplification and sequencing of the C3 AUG1/2 genomic DNA region. ∆2–25 A549 cells were created by transfection with dual pX459 plasmids targeting human C3 exon 2 and exon 25, resulting in excision of the entire intervening sequence, and testing resultant clones by PCR using flanking primers.

### Plasmids and site-directed mutagenesis

Full-length cDNA encoding human *C3* was cloned into pcDNA3 (Invitrogen). AUG to AUU and C3 STOP codon mutations in human *C3* sequence were introduced using the QuikChange II Site-Directed Mutagenesis Kit (Agilent Technologies), using primer pairs found in supplementary Table 1. Double and triple mutant C3-pcDNA plasmids were prepared from single (ΔAUG2 and ΔAUG3) or double (ΔAUG2 + 3) mutant constructs, respectively, using primer pairs which mutate the first AUG codon. All variants were confirmed by Sanger DNA sequencing (Eurofins Genomics). pET21a-BirA was purchased from Addgene (#20,857) and the BirA insert sub-cloned into pcDNA3. BAP-tagged C3 was generated by cloning of a synthesized C3-BAP fragment into WT C3-pcDNA3 using HindIII and BlpI restriction sites.

### Cell transfection and treatments

Transient transfection of HEK293 cells with C3 plasmids was accomplished using Lipofectamine 2000 (Invitrogen). After 2 days, medium was changed to OptiMem and cells further cultured for 2 days or treated after 1 day in OptiMem for overnight with 10 µM MG-132 (SelleChem #S2619) or 20 µM chloroquine (Sigma #C6628). Brefeldin A (ThermoFisher #B7450, 10 µg/ml) and diamide (Sigma #D3648) treatments were applied for 4 h before cell lysate collection. Cell lysate fractionation was prepared with the Mem-PER Plus Membrane Protein Extraction Kit (Invitrogen) according to the manufacturers’ instruction with minor modifications.

### SDS-PAGE and western blot

Fractionated cell lysates were separated by SDS-PAGE and transferred to a PVDF membrane using semi-dry blotting apparatus (BioRad). Membranes were blocked with Quench (50 mM Tris–HCl (pH 8.0), 150 mM NaCl, 0.1% Tween 20, 3% fish gelatin, Norland Products). C3 was detected using goat polyclonal anti-human C3 antibodies (Quidel #A304 and Calbiochem #204,869), or monoclonals detecting specific C3 epitopes.

### C3 ELISA

Maxisorp microtiter plates (96-well, Invitrogen) were coated with rabbit anti-human C3c (Dako #A0062) overnight, at 4 °C. After coating, plates were blocked with Quench for 1 h at 37 °C, then incubated with 50 µl/well with supernatant or lysate of WT or mutant C3 transfected HEK293 cells, diluted in Quench supplemented with 10 mM EDTA, for 1 h at 37 °C. C3 was detected using goat anti-human C3 antibody (Quidel #A304) and HRP-conjugated rabbit anti-goat immunoglobulins (Igs) (Dako #P0049) for 1 h at RT.

### Fluorescence microscopy

24 h after transfection with C3-GFP constructs, A549 cells were stained with AlexaFluor 488-labeled wheat germ agglutinin (ThermoFisher #W11261), washed, and imaged using Cytation 5 (BioTek Instruments). For confocal microscopy imaging, cells were additionally stained with Cytotracker ER red, mounted using ProLong Diamond Antifade Mountant with DAPI (Molecular Probes #P36971), and imaged using a LSM 510 Meta confocal microscope with Zen 2009 software (Zeiss).

### Detection of C3 by the BAP-BirA system

HEK293 cells were transiently transfected with BirA-pcDNA3 and WT or AUG1-codon mutated C3-BAP-pcDNA3 vectors. After 48 h, cells were collected, fractionated, C3 immunoprecipitated and eluted proteins run on SDS-PAGE under reducing condition with subsequent semi-dry Western blot. To detect C3 biotinylation, membranes were developed with HRP-conjugated streptavidin (R&D Systems #DY998).

### Bacteria

*Staphylococcus aureus* strain USA300 JE2 was grown in tryptic soy broth (Sigma) at 37 °C with 200 rpm shaking.

### Infection and collection of intracellular bacteria

Confluent A549 cells were washed with PBS to remove antibiotic-containing media and fresh OptiMem added to each well. Cells were infected with *S. aureus* at a multiplicity of infection (MOI) of 10:1. After 1 h, the cells were washed four times with PBS to remove unattached bacteria, and OptiMem containing 200 µg/ml of gentamycin (Sigma) added to kill uninternalized bacteria. After additional incubation, A549 cells were lysed using 1% saponin in PBS, and intact bacteria were collected by centrifugation (5000 g, 5 min) and washed two times in PBS to remove cell debris. A small volume from each sample was plated on blood agar to assess the number of bacteria recovered from different cell types.

### C3 deposition on bacteria from normal human serum and cytosol fraction

Log-phase *S. aureus* were washed twice in GVB +  + and resuspended in 100 µl Gelatin Veronal Buffer with Ca and Mg (GVB + +) containing either 1% Normal Human Serum (NHS) or 5% Pooled human liver cytosolic fractions (ThermoFisher #HMCYPL). Bacteria were incubated at 37 °C, with 800 rpm shaking for 30 min. Bacteria were washed twice in PBS. Bacteria were then incubated in PBS with 50 mM methylamine for 1 h at 37 °C, centrifuged for 5 min at 5000 g, and the supernatant containing dissociated C3 collected for C3 immunoprecipitation or trichloroacetic acid (TCA) protein precipitation. Collected C3 was resuspended in a small volume of PBS followed by addition of Laemmli sample buffer. Samples were boiled for 5 min before running on Western blot.

### Whole blood survival assay

Human blood was collected from healthy volunteers and treated with lepirudin (Refludan 50 µg/ml; Celgene). Approximately 1 × 10^5^ CFU of *S. aureus* were added to 475 µl of blood, and incubated rotating at 37 °C and 5% CO_2_. At various time points, samples were collected, serially diluted in PBS and plated on blood agar plates. After overnight incubation at 37 °C and 5% CO_2_, colonies were counted to assess bacteria survival. Alternatively, phagocytic HL60 cells were used instead of whole blood.

### Electron microscopy

4 h and 18 h, post-infection, A549 cells were washed with PBS, fixed, and mounted on electron microscopy grids. For each sample, approximately 50 cells were imaged in a FEI Tecnai biotwin 120 kV TEM. Analysis was performed blind by using the “shuffle and rename” function of Microscopy Image Browser software from the University of Helsinki [58].

### Statistical analyses

All data were analyzed using Prism software version 7 (GraphPad). Two-way and one-way ANOVA with multiple comparisons were used for statistical analyses as described in each figure legend. Differences with *p* < 0.05 were considered statistically significant and marked as *ns* > 0.05, *p < 0.05, **p < 0.01 and ***p < 0.001. Results indicate mean values, error bars SD and n represents individual repeats.

### Ethical permit

The full use of and collection of human blood for this study from informed healthy donors leaving written consent was approved by the ethical committee for Lund University (Dnr.2017/582). For extended methods, see supplementary information file.

### Supplementary Information

Below is the link to the electronic supplementary material.

Supplementary file1 (DOCX 2469 KB)

## Data Availability

All data are included in this article and its supplementary files.
