# Supplementary material for: Alternative translation and retrotranslocation of cytosolic C3 that detects cytoinvasive bacteria
Source: Cell Mol Life Sci. 2022 May 11;79(6):291. doi: 10.1007/s00018-022-04308-z (PMC9095555; doi:10.1007/s00018-022-04308-z)
Supplement: Supplementary file 1 — Supplementary file1 (DOCX 2469 KB) [file 18_2022_4308_MOESM1_ESM.docx]

**Supplementary Information for "Alternative translation and retrotranslocation of cytosolic C3 that detects cytoinvasive bacteria"**

Mariann Kremlitzka^1,2,3^, Lucie Colineau^1,3^, Alicja A. Nowacka^1^, Frida C. Mohlin^1^, Katarzyna Wozniak^1^, Anna M. Blom^1,4*^, Ben C. King^1,4^

^1^Lund University, Translational Medicine, 214 28 Malmö, Sweden

^2^Department of Immunology, Eötvös Loránd University, Budapest, Hungary

^3^these authors contributed equally to the study

^4^these authors jointly supervised the work

Corresponding author: Anna M. Blom

Email: [Anna.Blom@med.lu.se](mailto:xxxxx@xxxx.xxx)

**This PDF file includes:**

Supplementary text

Figures S1 to S3

Tables S1 to S2

Supplementary Information Text

**Extended Materials and Methods:**

**Cells and media**

HEK293 cells and the C3-expressing human epithelial cancer cell lines, A549 and Caco-2 were maintained in DMEM (Gibco) supplemented with 10% heat inactivated fetal bovine serum (FBS, Gibco). C3 knockout and first AUG codon edited A549 cells were generated with the CRISPR/Cas9 system, as described below. HL60 cells were grown in RPMI 1640 (Gibco), 10% FBS. All cells were tested regularly for mycoplasma contamination (Eurofins Genomics) and cultured at 37°C in 5 % CO_2._

**Creation of gene-edited A549 cells**

Guide RNA sequences targeting the canonical AUG (AGCACCATGGGACCCACCTC) and second AUG (AGAGTCATAACCACTCACAT) regions of the human C3 gene (NC_000019.10) were designed using the online CHOPCHOP algorithm^1^ and cloned into the pX459 plasmid^2^, a gift from Feng Zhang (Addgene plasmid #62988). For targeted mutagenesis of the second AUG site by homology directed repair (HDR), a single stranded DNA oligonucleotide incorporating a AUG/CTG mutation of the AUG site and silent mutation of the upstream PAM site was co-transfected with the guide RNA-containing pX459 plasmid. The repair template sequence for AUG2 was cc*atg*ggacccacctcaggtcccagcctgctgctcctgctactaacccacctccccctggctctggggagtGcG*Ctg*tgagtggttatgactctacccacaaacagggctggttctggggtggaagcagacatttgggggtc, where targeted mutations are underlined, and location of endogenous AUG sites 1 and 2 are in italics. After transfection, cells were selected using puromycin and individual clones were screened for C3 secretion and by amplification of the C3 AUG1/2 genomic DNA region using forward and reverse primers AAGGCAGGAGCCAGATAAAAAG and CAAATGCACCCTGAATTCTACA. PCR amplicons were sequenced using the same primers (Eurofins Genomics). Clones were identified with a homozygous indel mutation caused by non-homologous end joining repair at the predicted Cas9 cleavage site directly downstream of AUG1, causing a frameshift mutation with a predicted product of only 13 amino acids before a premature stop codon (∆AUG1). Clones were also identified with successful homozygous HDR-directed mutation of AUG2 (∆AUG2). C3-KO A549 cells were produced as previously described^3^. ∆2-25 A549 cells expressing the truncated C-terminus of the C3 α-chain were created by transfection with pX459 plasmids targeting human C3 sequences GGTGATGATAGAGTACCTGTCGG (exon 2) and GCTGGGTGCCCGTTTCACGAAGG (exon 25), and testing resultant clones by PCR using flanking forward and reverse primers, atccgtggaatgacaagcccactc and tctgagatccagagactgccatgtc. PCR amplicons were sent for sequencing to verify the exact genetic lesion.

**Plasmids and site-directed mutagenesis**

Full-length cDNA encoding human *C3* (NM_000064.3) was cloned into pcDNA3 vector (Invitrogen), referred as wild-type (WT) C3, and amplified in DH5α cells. Plasmids were purified with the Plasmid Plus Midi Kit from Qiagen. AUG to AUU and C3 STOP codon mutations in human *C3* sequence were introduced using the QuikChange II Site-Directed Mutagenesis Kit (Agilent Technologies), using primer pairs found in supplementary Table 1. Double and triple mutant C3-pcDNA plasmids were prepared from single (ΔAUG2 and ΔAUG3) or double (ΔAUG2+3) mutant constructs, respectively, using primer pairs which mutate the first AUG codon. C3 sequence containing STOP codon mutation was cloned to pcDNA3-EGFP vector (Addgene) using restriction enzymes XhoI and HindIII from Invitrogen. All variants were confirmed by Sanger DNA sequencing (Eurofins Genomics). pET21a-BirA with His-tag was purchased from Addgene and sub-cloned into pcDNA3. To prevent synthesis of His-tag, STOP mutation was introduced using site-directed mutagenesis (Agilent Technologies). BAP-tagged C3-pcDNA3 was generated by cloning of synthesized BAP with a part of C3 DNA sequence (InvivoGen) into WT C3-pcDNA3 after the signal peptide using HindIII and BlpI restriction sites. ΔAUG1-BAP-C3 was produced using the same method.

**Cell transfection and treatments**

Transient transfection of HEK293 cells with empty vector (mock), WT or mutant C3-pcDNA3 plasmid was accomplished using Lipofectamine 2000 reagent (Invitrogen). Cells were seeded on 12-well plates before transfection and grew until 90% confluency. On the day of transfection, DMEM+10% FBS was changed to OptiMem (Gibco) and cells were transfected with 3 µg plasmid complexed with 3 µL Lipofectamine 2000 reagents. After transfection (8h), cells were washed into DMEM+10% FBS and further cultured for 2 days in DMEM+10% FBS. After 2 days, medium was changed to OptiMem and cells further cultured for 2 days or treated after 1 day in OptiMem for overnight with 10 µM MG-132 (SelleChem #S2619) or 10 µM chloroquine (Sigma #C6628). Brefeldin A (ThermoFisher #B7450, 10 µg/ml) and diamide (Sigma #D3648) treatments were applied for 4 h before cell collection. After incubation, cell supernatant was collected and cells pelleted for lysis and Western blot analysis.

**In vitro transcription and translation of human C3**

To analyze the effect of START codon mutations on C3 expression in cell-free environment, *in vitro* translation was carried out according to the manufacturers’ instructions (Promega). Briefly, 1 µg WT or mutant C3-pcDNA3 plasmids were diluted into 40 µL TNT® T7 Quick Master Mix, supplemented with 20 µM methionine, 1 µl T7 TNT PCR Enhancer and 1 µl canine pancreatic microsomal membranes (Promega) in a final volume of 50 μl. Sample was incubated at 30°C for 90 minutes and run on 4-10% gradient SDS-PAGE.

**Cell lysate preparation and fractionation**

Cell lysates and fractionation of A549 cells (5*10^6^/ sample) and pcDNA3-C3 transfected HEK293 cells (12-well plate, combined cells of the wells) were prepared with the Mem-PER Plus Membrane Protein Extraction Kit (Invitrogen) according to the manufacturers’ instruction with minor modifications. Cell pellets were re-suspended in 400 µl permeabilization buffer and incubated for 10 min on ice. The resulting lysates were then centrifuged for 10 min at 16.000 *g*, supernatants containing cytoplasmic proteins collected and stored at –20°C until further use. After washing 3X in PBS, the pellet was re-suspended in 260 µl solubilization buffer and incubated for 30 min on ice. The resulting lysates were then centrifuged for 15 min at 16.000 *g*, supernatants referred as membrane fraction collected and stored at –20°C until further use. For measurement of C3 in lysates using ELISA, transfected HEK293 cells were lysed using 50 mM Tris pH 7.4, 150 mM NaCl, 1% NP40, 0.5% sodium deoxycholate, supplemented with protease and phosphatase inhibitors, for 15 min on ice.

**Immunoprecipitation**

To analyze glycosylation and ubiquitinylation of C3, the protein was immunoprecipitated. To this end, cytoplasmic or membrane fractions of transfected HEK293 was pre-cleared with 10 µl protein A/G coated magnetic beads (Invitrogen) for 1 h at 4°C. Thereafter, 10 µg anti-C3c antibody (Dako, #A0062) was added to the lysates, incubated overnight at 4°C, supplemented with 20 µl / sample protein A/G coated magnetic beads for the last 2 h. Beads were washed 3X in PBS and 1X in PBS containing 300 mM NaCl. Antibody-C3 complexes were eluted by boiling the samples for 10 min with 30 µl Laemmli buffer containing 25 µM DTT (Saveen Werner). Eluted proteins were run on SDS-PAGE and developed by Western blot with HRP-conjugated Concanavalin A (Invitrogen, glycosylation), or with the monoclonal mouse anti-ubiquitin (Cell Signaling Technology, clone P4D1, ubiquitinylation) or with the polyclonal goat anti-human C3 (Quidel, #A304) antibodies as described below.

**SDS-PAGE and Western blot**

The fractionated cell lysates were separated by SDS-PAGE under reducing (25 mM DTT) or non-reducing conditions and transferred to a PVDF membrane using semi-dry blotting apparatus (BioRad). The membranes were blocked with Quench solution (50 mM Tris–HCl (pH 8.0), 150 mM NaCl, 0.1% Tween 20, 3% fish gelatin, Norland Products) or in PBS containing 0.1 % Tween 20 for 1 h at room temperature (RT) and incubated with the primary antibodies overnight at 4°C. C3 was detected using goat polyclonal anti-human C3 antibodies (Quidel, #A304 and Calbiochem, #204869). As loading controls, rabbit anti- β-tubulin (Abcam, #ab6046, cytoplasmic marker), rabbit anti- β-actin (Sigma, A2066), rabbit anti-Na/K ATPase (Abcam, #ab76020, membrane marker) or rat anti-human calnexin (BioLegend, #699402, membrane marker) antibodies were used. When testing for specific C3 epitopes, the following antibodies were used: mouse monoclonal anti-C3a (Hycult #HM2073), rabbit polyclonal anti-C3d (Dako #A0063), mouse monoclonal Anti-C3 / C3b antibody (which we termed anti-C3 alpha’2, Abcam #ab11871), rabbit monoclonal anti-C3 (termed anti-C3 C-term, Abcam #ab200999). Proteins were visualized using polyclonal HRP-conjugated antibodies against rabbit, rat, mouse or goat immunoglobulin (Dako) diluted in Quench. After incubation with primary and secondary Antibodies, membranes were washed four times with Immunowash (50 mM Tris–HCl (pH 8.0), 150 mM NaCl, 0.1% Tween 20). Membranes were developed by the enhanced chemiluminescence (ECL) method (Millipore) and pictures were captured with the ChemiDoc MP system using Image Lab software from Bio-Rad.

**Detection of C3 glycosylation**

Glycosylation of cytoplasmic C3 was investigated either by immunoprecipitation of C3 from cytoplasmic fraction of HEK293 cells and Western blotting with HRP-conjugated Concanavalin A (#L6397, dilution 1000X in Quench) or by treatment of cytoplasmic lysates with PNGase F under non-reducing condition (New England Biolab). Briefly, 20 µg total protein content of cytoplasmic fractions was combined with 2 µl of GlycoBuffer 2 (10X) and final volume adjusted to 20 µl with distilled water. Deglycosylation was induced by addition of 4 µl PNGase F (2000U) and carried out at 37°C for 18 h. As positive controls, 5 µg purified C3 was deglycosylated in the same reaction or left untreated. Glycosylation state of C3 was investigated by SDS-PAGE and Western blotting with the polyclonal anti-C3 antibody from Quidel.

**Raising of antibodies recognizing unglycosylated C3**

Rabbits were immunized using peptide epitopes covering the N-glycosylation sites of human C3 α- and β-chains (PEGIRMNKTVAVR and ATNHMGNVTFTIPANR). Blood was drawn and the IgG fraction purified on protein A columns, followed by affinity purification on peptide-coupled columns. Antibodies recognizing glycosylated C3 were then absorbed on a column coupled with serum-purified C3. Resultant antibodies from two of four rabbits showed specific detection of deglycosylated C3 by Western blot. Vaccination, animal care, and antibody purification were carried out by Capra Science Antibodies AB (Ängelholm, Sweden).

**C3 ELISA**

C3 level in the secreted supernatants or lysates of HEK293 cells was investigated by sandwich ELISA. Maxisorp microtiter plates (96-well, Invitrogen) were coated with rabbit anti-human C3c (Dako, #A0062, dilution: 1000X in PBS (Medicago AB)) overnight, at 4°C. Between each of the following steps, the plates were washed four times with Immunowash. After coating, plates were blocked with Quench for 1 h at 37°C and incubated with 50 µl/well supernatant or lysate of WT or mutant C3 transfected HEK293 cells, diluted in Quench supplemented with 10 mM EDTA, for 1 h at 37°C. Purified C3, purchased from Complement Technologies, was used as a standard (range: 500-7.8 ng/ml). After incubation, bound proteins were detected using goat anti-human C3 antibody (Quidel #A304, dilution: 4000X in Quench) and HRP-conjugated rabbit anti-goat immunoglobulins (Igs) (Dako, #P0049, dilution: 2000X in Quench,) for 1 h at RT. As substrate, TMB ONE (Kementec) was used (absorbance 450 nm).

**Fluorescence imaging**

To analyze intracellular distribution of WT or AUG mutant C3 variants, A549 cells were transfected with the WT or ∆AUG1 mutated C3-pcDNA3 GFP constructs as described above. After 24 h incubation, cells were stained with wheat germ agglutinin conjugated to AlexaFluor 488 (ThermoFisher #W11261), washed, and fluorescence imaged using Cytation 5 (BioTek Instruments). For confocal microscopy imaging, cells were additionally stained with Cytotracker ER red, and mounted using ProLong Diamond Antifade Mountant with DAPI (Molecular Probes # P36971), and imaged using a LSM 510 Meta confocal microscope with Zen 2009 software (Zeiss).

**Detection of C3 retrotranslocation by the BAP-BirA system**

To investigate retrotranslocation and prove presence of C3 in the cytoplasm, HEK293 cells were transiently transfected with BirA-pcDNA3 and WT or canonical START site mutated C3-pcDNA3 vectors. After 48 h, cells were collected, fractionated, C3 immunoprecipitated using protocols described above and eluted proteins run on SDS-PAGE under reducing condition with subsequent semi-dry Western blot. To detect biotinylation of C3, membranes were developed with HRP-conjugated streptavidin (R&D Systems, #DY998). The presence of C3 in the eluted fractions was visualized using the goat anti-human C3 Ab from Quidel.

**Quantitative PCR**

RNA was extracted using RNeasy Plus Mini Kit (Qiagen) and cDNA was synthesized using oligo-dT primers and SuperScript III (Invitrogen). Quantitative PCR was performed with Taqman qPCR assay from Applied Biosystems (Human C3 #Hs00163811_m1 and Hs99999909_m1) on a Viia7 Real-Time PCR system (ThermoFisher). Expression levels of C3 were calculated after normalization with the geometric mean of the housekeeping gene hypoxanthine-guanine phosphoribosyltransferase (HPRT).

**Bacteria**

*S. aureus* strain USA300 JE2 was grown in tryptic soy broth (Sigma) at 37°C with 200 rpm shaking. After overnight growth, cultures were diluted to OD600nm = 0.1 and grown under the same conditions until they reached exponential phase (OD600nm = 0.3-0.4). Bacteria were centrifuged, washed once in sterile PBS and adjusted to OD600nm = 1. For CFU counting, *S. aureus* were plated on blood agar plates.

**Infection and collection of intracellular bacteria**

Confluent A549 cells were washed with PBS to remove antibiotics-containing media and fresh OptiMem was added to each well. Cells were infected with *S. aureus* at a multiplicity of infection (MOI) of 10:1. After 1 h infection, the cells were washed four times with PBS to remove unattached bacteria, and OptiMem containing 200 µg/ml of gentamycin (Sigma) was added to kill uninternalized bacteria. Infected cells were incubated at 37°C, 5% CO_2_ for 24 h. A549 cells were lysed using 1% saponin in PBS, intact bacteria were collected by centrifugation (5000g, 5 min) and washed two times in PBS to remove cell debris. A small volume from each sample was plated on blood agar to assess the number of bacteria from different cell types.

**C3 deposition on bacteria from normal human serum and cytosol fraction**

Log-phase *S. aureus* were washed twice in GVB++ and resuspended in 100 µl Gelatin Veronal Buffer with Ca and Mg (GVB++) containing either 1% Normal Human Serum (NHS) or 5% Pooled human liver cytosolic fractions (ThermoFisher HMCYPL). Human blood and serum from informed consenting healthy donors was collected under ethical permit Dnr.2017/582. Bacteria were incubated at 37°C, with 800 rpm shaking for 30 min. Bacteria were washed twice in PBS.

**Assessing C3 deposition on bacteria**

Bacteria were incubated in PBS with 50 mM methylamine for 1 h at 37°C, with shaking at 800 rpm to detach C3 fragment bound to their surface. Bacteria were centrifuged for 5 min at 5000g, and the supernatant containing dissociated C3 was collected to undergo immunoprecipitation as described above, or trichloroacetic acid (TCA) protein precipitation.

TCA (20%, Sigma) was added to each sample, which was vortexed and spun down. Samples were incubated on ice for 30 min then centrifuged at 14,000g for 15 min at 4°C to pellet the precipitated proteins. Supernatant was removed, and the pellets were washed with ice-cold acetone. Once dried, the pellets were resuspended in a small volume of PBS followed by addition of Laemmli sample buffer. Samples were boiled for 5 min, and sonicated for 5 seconds.

**Whole blood survival assay**

Human blood was collected from healthy volunteers and treated with lepirudin (Refludan 50 µg/ml; Celgene). Approximately 1x10^5^ CFU of *S. aureus* were added to 475 µl of blood, and blood suspensions were incubated on an end-over-end rotator at 37°C and 5% CO_2_. At various time points, a sample of the suspension was collected, serially diluted in PBS and plated on blood agar plates. After overnight incubation at 37°C and 5% CO_2_, colonies were counted to assess bacteria survival.

**Killing of bacteria by neutrophil-like HL60 cells**

HL60 cells were differentiated with 1.25% DMSO. After 6 days, the cells were collected, counted and resuspended in PBS. 0.33 x10^6^ differentiated HL60 cells were combined with approximately 5 x10^5^ *S. aureus*, and incubated on an end-over-end rotator at 37°C and 5% CO_2_. At each timepoint, a sample of suspension was collected, serially diluted in PBS and plated on blood agar plates to assess bacteria survival.

**Analysis of bacteria with flow cytometry**

Bacteria were fixed in 4% paraformaldehyde for 15 min and washed in PBS. Bacteria were incubated with BODIPY FL-conjugated Vancomycin (1 µg/ml, Invitrogen # V34850), and rabbit anti-goat IgG Alexa Fluor 647 (1:1000, Invitrogen #A21446) to detect SpA, for 30 min at RT. Bacteria were washed once in PBS and resuspended in PBS for analysis using a CytoFLEX flow cytometer.

**PCR for staphylococcal virulence factors**

Intracellular bacteria were collected as described above and washed once in PBS. Bacteria were centrifuged and pellets were stored at -80°C. Pellets were resuspended in TE buffer containing 100 µg/ml lysostaphin (Sigma #L7386) and 15 mg/ml lysozyme and incubated for 10 min at 37°C with shaking at 1400 rpm. RNA was extracted with the ReliaPrep RNA Miniprep Kit (Promega, #Z6011). Equal amounts of RNA were used as template for reverse transcription PCR using Superscript IV Reverse Transcriptase (Invitrogen #18090050) and random hexamer primers. PCR reactions were performed using newly generated cDNA, DreamTaq green MasterMix (ThermoFisher #EP0705), and primers listed in supplementary table 2. PCR reactions were then loaded onto a 2% agarose gel. Band intensity was measured, and data was normalized to 16S and input bacteria.

**Cleavage of C3 by *S. aureus* proteases**

Serum-purified C3 (180 ng, final concentration of 50 nM) was added per tube diluted into 10 µl of 20 mM HEPES, pH 7.5 with 150 mM NaCl. 10 µl of the same buffer was added, containing titrations of either V8 or ScpA for a final concentration of 1000, 250, 60, 15 and 4 nM. V8 and ScpA were produced as previously described^4^. Controls with only C3 or only proteases were also prepared. Samples were incubated at 37°C for 1 h, boiled in reducing Laemmli buffer, separated by electrophoresis and blotted.

**Electron Microscopy**

A549 cells were infected with *S. aureus* as described above. After 4 h and 18 h, infected cells were washed with PBS, detached using trypsin, and washed twice in PBS. Cells were incubated with fixative buffer (4% paraformaldehyde in 0.1 M Sorensen phosphate buffer – 133 mM NaH_2_PO_4_ + 133m M KH_2_PO_4_) for 1 h at RT. Cells were then centrifuged, washed in 0.1 M phosphate buffer, centrifuged, and pellets were stored in 0.1 M phosphate buffer at 4°C. Cell pellets were embedded in Agarose type VII, postfixed in 1% osmium tetroxide in 0.1 M Sorensen phosphate buffer, dehydrated in rising concentration of acetone, followed by 1:1 mix polybed-aceton, embedded in pure Polybed 812. Polymerised blocks were sectioned by Leica UM7 ultrotome. The sections were mounted on a pioloform coated copper Maxtafom H5 grid. The sections were contrasted with 4% Uranyl acetate followed with 1% lead citrate, and analyzed in a FEI Tecnai biotwin 120 KV TEM.

For each sample, approximately 50 cells were imaged, at a high magnification to count bacteria and assess their location (cytosolic or vacuolar). This analysis was performed blind by using the “shuffle and rename” function of Microscopy Image Browser software from the University of Helsinki^5^.

**Statistical analyses**

All data were analyzed using Prism software version 7 (GraphPad). Two-way and one-way ANOVA with multiple comparisons were used for statistical analyses as described in each figure legend. Differences with p < 0.05 were considered statistically significant and marked as ns>0.05, *p < 0.05, **p < 0.01 and ***p < 0.001. Results indicate mean values, error bars SD and n represents individual repeats.

Fig. S1.

S1: Mapping of the C-terminal C3 fragment deposited onto intracellular bacteria.

The deposited product was not recognized by antibodies against the glycosylation sites on α- or β- chains, or by anti-C3a, or anti-C3d monoclonals. As control, the product was proven to be present on membranes by detection with anti-C3 polyclonal antibody. The product was also detected by anti-C3 a'2 and anti-C-terminal monoclonals, mapping the product to the C-terminal region of mature C3 α-chain. Antibody recognition epitopes are shown in panel B.

Fig. S2.


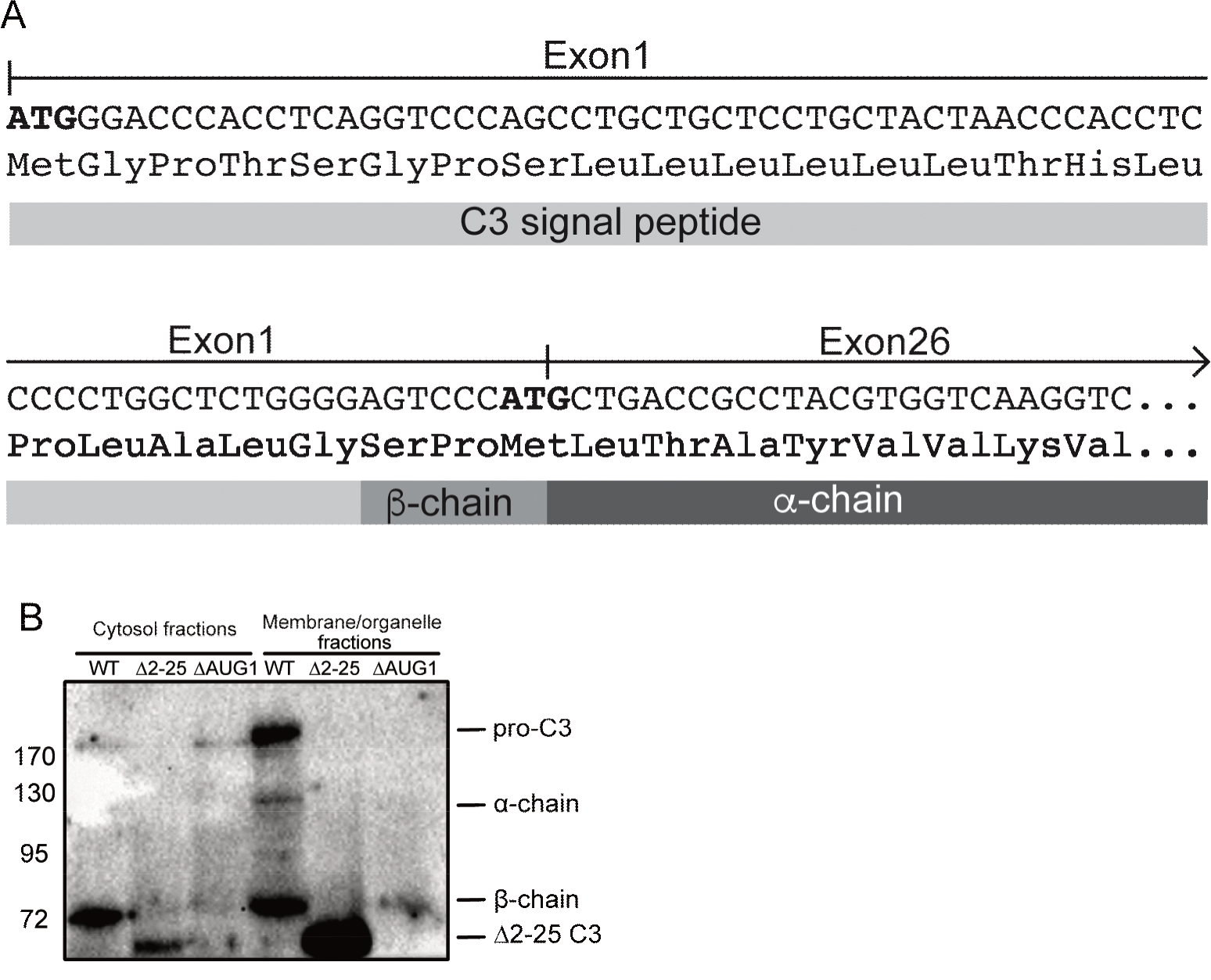


S2: A) Confirmed sequence of the truncated C3 product expressed in ∆2-25 A549 cells. Paired guide RNAs cleaved within intron 1 and exon 25, removing the intervening genomic sequence, as confirmed by PCR and sequencing. Subsequent sequencing of cDNA confirmed that exon 1 is spliced directly to the splice acceptor site of exon 26, which does not result in a frameshift of the coding sequence, but leaves only 3 amino acids of the β-chain in the expressed protein, and removes the first 403 amino acids from the α-chain. G is the first nucleotide in both exons 2 and 26, leaving the first methionine-encoding AUG codon at the boundary of exon 1 and either 2 or 26 (AUG2) unchanged. A comparative map of the entire 67 kDa truncated protein is found in figure 6B. B) Western blot with an anti-C3 polyclonal antibody of ∆2-25 A549 cell cytosolic and membrane/organelle fractions, with WT and ∆AUG1 A549 cells as comparisons, showing presence of the roughly 65 kDa C-terminal C3 fragment within the cytosol.

Fig. S3.


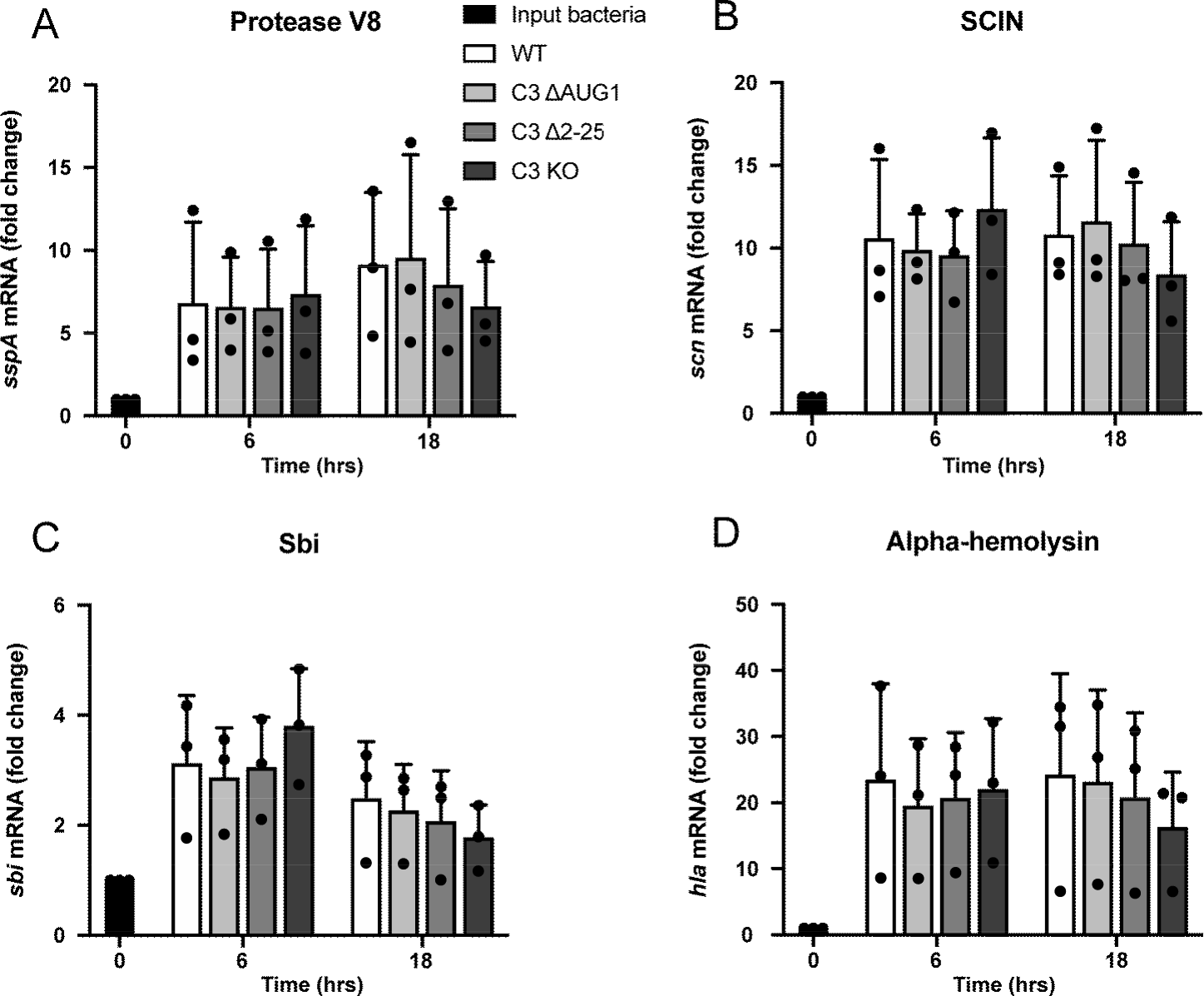


S3: mRNA levels for the *S. aureus* virulence factors V8, SCIN, Sbi and Hla are similar in bacteria from WT, ∆AUG1, ∆2-25, and C3KO A549 cells. Intracellular bacteria were collected by lysis of A549 cells at 6 and 18 h post-infection. RNA was extracted, reverse-transcribed and PCR was performed using primers for *sspA* (V8), *scn* (SCIN), *sbi*, *hla* and 16S. mRNA levels are represented normalized to 16S for each sample, and input bacteria for each of the three independent experiments.

**Supplementary Table 1** Primers used to mutate C3 with site-directed mutagenesis

| Mutation | | |  |
| --- | --- | --- | --- |
| Name/type | Nucleotide position in cDNA | Amino acid position in pro C3 | Forward primers (5’ to 3’)* |
| First (canonical) AUG codon | c.96G>T | 1 | 5’ GCACTGTCCCAGCACCAUUGGACCCACCTCAGGTC 3’ |
| Second AUG codon | c.168G>T | 25 | 5’ CTGGCTCTGGGGAGTCCCAT**T**TACTCTATCATCACC 3’ |
| Third AUG codon | c.219G>T | 42 | 5’ GGAGAGCGAGGAGACCAT**T**GTGCTGGAGGCCCACG 3’ |
| STOP codon | c.5083T>G |  | 5’ TCTTTGGGTGCCCCAAC**G**GACCACACCC 3’ |

* Mutations are marked in boldface and underlined.

**Supplementary Table 2** Primers used in PCR for *S. aureus* virulence factors

| Genes | Protein | Primers sequences (forward and reverse) | Reference |
| --- | --- | --- | --- |
| *16S rRNA* |  | GTAGGTGGCAAGCGTTATCC  CGCACATCAGCGTCAG | Chu et al.^6^ |
| *spa* | Staphylococcal protein A (SpA) | CAAACCTGGTCAAGAACTTGTTGTTG  GCTAATGATAATCCACCAAATACAGTTG | Garzoni et al.^7^ |
| *sspA* | Serine protease V8 (or SspA) | CTTATATTCAAGTTGAAGCACCTACTGG  CTTTTAAAGCATGAGGATCACCGTG | Garzoni et al.^7^ |
| *scn* | Staphylococcal complement inhibitor (SCIN) | CTTGCCAACATCGAATGAATATCAAAAC  GTCTTTTGACTTAAGAGCATACATTGC | Garzoni et al.^7^ |
| *sbi* | Immunoglobulin-binding protein Sbi | GAAGAACAACGTAACCAATACATCAAAAC  GTAAAAAGCGTTTTGTTGTGCAACAC | Garzoni et al.^7^ |
| *hla* | Alpha-hemolysin (Hla) | ATGAGTACTTTAACTTATGGATTCAACGG  AGTGTATGACCAATCGAAACATTTG | Garzoni et al.^7^ |

**Supplementary References:**

1 Labun, K., Montague, T. G., Gagnon, J. A., Thyme, S. B. & Valen, E. CHOPCHOP v2: a web tool for the next generation of CRISPR genome engineering. *Nucleic Acids Res* **44**, W272-276, doi:10.1093/nar/gkw398 (2016).

2 Ran, F. A. *et al.* Genome engineering using the CRISPR-Cas9 system. *Nat Protoc* **8**, 2281-2308, doi:10.1038/nprot.2013.143 (2013).

3 King, B. C. *et al.* Complement Component C3 Is Highly Expressed in Human Pancreatic Islets and Prevents beta Cell Death via ATG16L1 Interaction and Autophagy Regulation. *Cell Metab* **29**, 202-210 e206, doi:10.1016/j.cmet.2018.09.009 (2019).

4 Jusko, M. *et al.* Staphylococcal proteases aid in evasion of the human complement system. *J Innate Immun* **6**, 31-46, doi:10.1159/000351458 (2014).

5 Belevich, I., Joensuu, M., Kumar, D., Vihinen, H. & Jokitalo, E. Microscopy Image Browser: A Platform for Segmentation and Analysis of Multidimensional Datasets. *PLoS Biol* **14**, e1002340, doi:10.1371/journal.pbio.1002340 (2016).

6 Chu, A. J. *et al.* Nusbiarylins Inhibit Transcription and Target Virulence Factors in Bacterial Pathogen Staphylococcus aureus. *Int J Mol Sci* **21**, doi:10.3390/ijms21165772 (2020).

7 Garzoni, C. *et al.* A global view of Staphylococcus aureus whole genome expression upon internalization in human epithelial cells. *BMC Genomics* **8**, 171, doi:10.1186/1471-2164-8-171 (2007).
